# The evolution of gene functional repertoire in Amorphea: divergent strategies across Amoebozoa, Fungi, and Metazoa

**DOI:** 10.1093/molbev/msag071

**Published:** 2026-05-20

**Authors:** Alex Gàlvez-Morante, Cédric Berney, Daniel J Richter

**Affiliations:** Institut de Biologia Evolutiva (CSIC-Universitat Pompeu Fabra), Passeig Marítim de la Barceloneta 37-49, Barcelona 08003, Spain; Institut de Biologia Evolutiva (CSIC-Universitat Pompeu Fabra), Passeig Marítim de la Barceloneta 37-49, Barcelona 08003, Spain; Institut de Biologia Evolutiva (CSIC-Universitat Pompeu Fabra), Passeig Marítim de la Barceloneta 37-49, Barcelona 08003, Spain

**Keywords:** Amoebozoa, Metazoa, Fungi, evolution, Amorphea, ancestral reconstruction, COG, Pfam

## Abstract

Metazoa and Fungi have been extensively studied to reconstruct the trajectory of Opisthokont evolution. Their sister group, Amoebozoa, provides additional potential to generate valuable insights into the origins of Opisthokont lineages. Amoebozoa represent a diverse group of amoeboid organisms, which have adapted to a wide range of environments and ecological niches. Studying Amoebozoa not only helps to illuminate Opisthokont evolution but also reveals the mechanisms that have driven amoebozoan ecological success.

Here, we report the discovery of *Apostamoeba explorator* strain BEAP0066, representing a novel lineage within Amoebozoa with intriguing behaviors like the “double-amoeba,” a behavior characterized by the bipolarization of a cell into two poles that coexist and act as two semi-independent cells.

By analyzing the gene content of *A. explorator* and diverse amoebozoans with ancestral gene content reconstructions, correspondence analyses of Clusters of Orthologous Groups category composition and protein families database (Pfam) clan clustering, we revealed distinct evolutionary trajectories for Amoebozoa, Metazoa, and Fungi. Amoebozoa retained an ancestral Amorphea-like state, characterized by an enrichment of genes related to motility, phagocytosis, and rapid cellular response, while Metazoa specialized in multicellularity-related genes and Fungi in metabolism and transport. These findings suggest that retention of gene function composition, rather than gene loss, played a key role in shaping Amoebozoa evolution.

Significance statement Cells of species within the lineage Amoebozoa are capable of maintaining a dynamically stable identity and move by extending temporary cytoplasmic projections, usually composed of actin, called pseudopodia. The study of amoebae is crucial for advancing research in medicine, ecology, and evolution. Understanding the phylogeny of Amoebozoa remains a key focus in phylogenomics, as deep divergences within the group complicate the taxonomic placement of certain taxa and have implications for character evolution. Thus, the discovery, identification, and characterization of new Amoebozoa species can help resolve current uncertainties. Recent studies have identified divergent trajectories in gene content composition within one lineage of Amorphea, the Opisthokonts. Fungi evolved through an expansion of metabolic genes, whereas Metazoa accumulated genes associated with multicellularity. Inspired by these findings, we analyzed the proteomes of Amoebozoa, Metazoa, and Fungi. Using correspondence analysis of the relative composition of Clusters of Orthologous Groups (COG), ancestral reconstruction and the analysis of protein families database (Pfam) domain clan presence in supergroup-specific gene clusters, we aimed to determine whether the three supergroups within Amorphea exhibit distinct clustering patterns. Our results provide evidence of divergent functional evolution in Amoebozoa, Fungi, and Metazoa. Amoebozoa retained an ancestral Amorphea-like state, characterized by an enrichment of genes related to motility, phagocytosis, and rapid cellular response; while we recover results consistent with previous studies for Metazoa and Fungi. These findings suggest that retention of gene function composition, rather than gene loss, played a key role in shaping Amoebozoa evolution.

## Introduction

Cells of species within the lineage Amoebozoa are capable of maintaining a dynamically stable identity and move by extending temporary cytoplasmatic projections, usually composed of actin, called pseudopodia. Amoebozoa is the single largest taxon composed almost exclusively of amoeboid organisms and is one of the two major lineages within the supergroup Amorphea, together with Opisthokonta (the group that includes Fungi and Metazoa) (Adl et al. 2019; Tekle et al. 2022). Amoebozoans populate virtually all of Earth's environments, from freshwater (Page 1988; Mesentsev 2025) to marine water (Page 1983), soil (Smirnov and Brown 2004), and even other organisms (Dyková and Lom 2004). The prevalence of this lifestyle is evidence of the evolutionary success of the Amoebozoa and their ability to adapt to different environmental conditions and ecological roles.

The study of Amoebozoa is crucial for contributing to knowledge in varied fields including medicine, as they are part of our microbiome (Stensvold et al. 2023) and pathogens (Usuda et al. 2022); ecology, as amoebae occupy a wide variety of ecological niches, acting as predators (Geisen et al. 2015) and parasites (Padrós and Constenla 2021); or evolution, as their phylogenetic position makes them a relevant outgroup to study the evolution of Opisthokonta (Tekle et al. 2022).

The main amoebozoan cell form is locomotive (the active and feeding cell), but they are able to undergo a variety of transitions among forms or stages in the course of their life cycle. One widespread example would be the cyst, a dormant life stage generated to resist harsh environmental conditions, but many are able to generate other life stages, including flagellates and spores, among other reproductive and social behaviors (Schilde and Schaap 2013). Reconstructing the phylogenetic relationships within Amoebozoa is an active topic in the phylogenomics field, as deep divergences make it difficult to produce robust placement of some key taxa, with implications for character evolution in the group (Tekle et al. 2022). Thus, the discovery, identification, and characterization of new Amoebozoa species can contribute to resolving current uncertainties.

Recently, divergent trajectories in gene content composition were identified in a comparison of the evolution of the two major lineages within Opisthokonts: Fungi, whose evolution was characterized by an expansion of metabolic genes, and Metazoa, which preferentially accumulated genes potentially important for their multicellularity (Ocaña-Pallarès et al. 2022). These findings inspired us to expand this analysis to a large sampling of amoebozoans, alongside Metazoa and Fungi, by performing correspondence analysis (CA; a statistical method for visualizing relationships between rows and columns in a contingency table, similar to principal component analysis but suited for compositional data) of the relative composition of Clusters of Orthologous Groups (COG; functional categories of proteins grouped based on orthology that are thought to represent conserved functional roles across species), replicating a similar methodology to the one implemented in Ocaña-Pallarès et al., and allowing for the discovery of patterns of gene function composition among species in the three groups, and the determination of whether Amoebozoa may have followed a distinct path of gene function composition in comparison to animals and fungi.

In order to enhance our analyses of Amoebozoa diversity and gene content, we incorporated two recently isolated amoebozoan species from our laboratory cultures, *Vannella septentrionalis* and an unidentified amoebozoan with strain designation BEAP0066. Remarkably, BEAP0066 turned out to represent a phylogenetically novel lineage, allowing us to contribute to knowledge of Amoebozoa diversity due to its evolutionary position and substantial divergence from previously described groups. We begin by characterizing this new isolate, starting with its phylogenetic placement within Amoebozoa followed by its morphological and biological features, through a combination of microscopy techniques and phylogenetic approaches. Next, we incorporate both new amoebozoan isolates as additional taxa in the analyses we present of gene composition within Amorphea. Our analyses provide evidence of divergent evolution of gene function composition among Amoebozoa, Fungi, and Metazoa. Additionally, we conducted an ancestral reconstruction of gene family evolution across Amorphea to compare the COG profiles of ancestral species with those of extant supergroups. To complement these analyses, we identified overrepresented Pfam domain clans in gene clusters specific per Amoebozoa, Fungi, and Metazoa to better understand the changes in functional potential that drove the evolution of Amorphea.

## Results

### Phylogenetic placement of newly isolated Amoebozoa strains

In this study, we isolated two new amoeba strains in culture, BEAP0066 and BEAP0079. One strain, BEAP0079, displayed morphological characteristics matching the genus *Vannella*. Its 18S small subunit ribosomal RNA gene sequence is 99.1% identical to the GenBank sequence EF051197 of the type strain of *Vannella septentrionalis* (CCAP 1589/10). This sequence identity is well within the range of possible intra-strain variability that has been observed among clonal cultures of species within the genus *Vannella* (Nassonova et al. 2010), justifying its identification as *V. septentrionalis*. The other strain, BEAP0066, did not match the morphology or 18S rRNA sequence of any known species. We described strain BEAP0066 as *Apostamoeba explorator* gen. et sp. nov., and determined its position within Amoebozoa via phylogenetic reconstruction.

**Figure 1:**
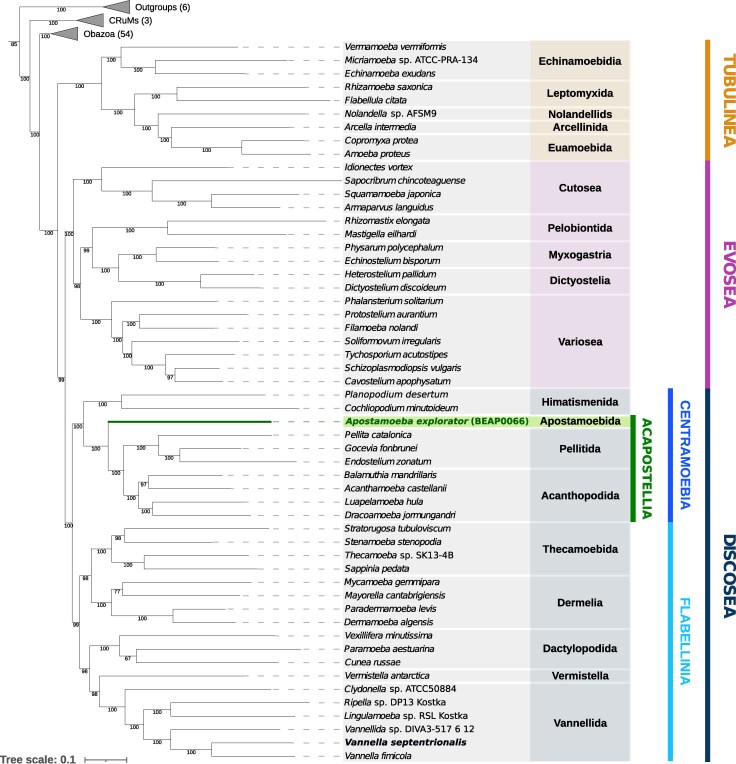
*Apostamoeba explorator* and *Vannella septentrionalis* phylogenetic position within Amoebozoa. Phylogenetic tree of Amoebozoa diversity based on 230 genes, with new sequences from BEAP0066 and *V. septentrionalis*, reconstructed with PhyloFisher and IQ-TREE using the LG+R10 model of sequence evolution. Node support reflects 1000 bootstraps. *Apostamoeba explorator* branches with full support within Discosea as a sister to Pellitida + Acanthopodida. *V. septentrionalis* branches as sister to *Vannella fimicola*, as expected. Number of species included in each outgroup is indicated in parenthesis. See File S12 for the phylogenetic tree with all outgroup species represented. The tree was manually rooted between Amorphea + CRuMs and Outgroups (containing Malawimonadidae, Diaphoretickes and Ancyromonadida).

*A. explorator*'s 18S small subunit ribosomal RNA gene sequence is highly divergent and was placed as a very long branch, with low bootstrap support, as sister to the centramoebid lineages Acanthopodida and Pellitida (Figure S1). To resolve the phylogenetic position of *A. explorator* within Amoebozoa, we made use of its transcriptome and reconstructed a phylogenomic tree with PhyloFisher (Tice et al. 2021) followed by IQ-TREE (Nguyen et al. 2015) (Fig. 1). Our new isolate branches inside Discosea, sister to Pellitida and Acanthopodida (*V. septentrionalis* recovered an expected placement inside Vannellida). The independent position of *Apostamoeba* compared to known amoebozoan orders demonstrates that it represents a new order-level lineage, that we named Apostamoebida ord. nov. Additionally, a total of 62 V4 ASVs (amplicon sequence variants) belonging to Apostamoebida were identified in the EukBank database by a blastn search using the V4 region of *A. explorator* as a sequence query, together representing diversity within Apostamoebida but outside currently described amoebozoan lineages (Figure S2).

### Morphology and behavior of *Apostamoeba explorator*

**Figure 2:**
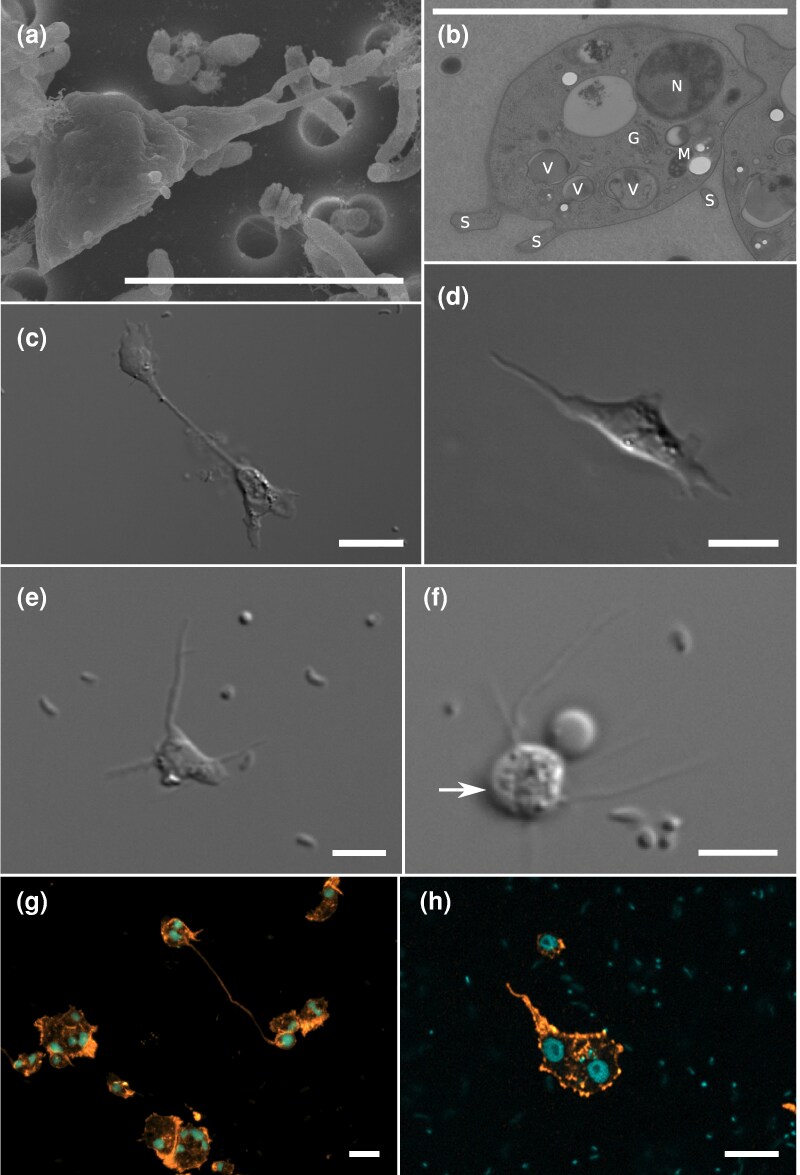
Morphology and ultrastructure of *Apostamoeba explorator*. a) Scanning electron microscopy (SEM) of a single locomotive cell with multiple morphologically diverse prey bacteria. Extra SEM images are provided in File S13. b) Transmission electron microscopy (TEM) of a locomotive cell (N = Nucleus, V = Vacuole, S = Subpseudopodia, M = Mitochondrion, G = Golgi). Extra TEM images are provided in File S14. c) Differential interference contrast (DIC) image of the “double-amoeba” morphology (bipolarization of a cell and the generation of two poles that coexist and act semi-independently). d and e) DIC images of a locomotive cell (see video S1 for multiple instances of locomotive form behavior and movement). f) DIC image of floating form. g and h) Immunoﬂuorescence images stained with Phalloidin 488 (actin = orange) and Draq-5 (nuclei = cyan), showing multinucleate locomotive cells and the double-amoeba morphology. Scale bars in all panels = 5 μm.

We identified numerous cell type morphologies in *A. explorator* cell cultures (Fig. 2, Figure S3). *A. explorator* locomotive cells (Fig. 2d and e) possess a very thin glycocalyx and present a variable cell length, which (excluding subpseudopodia) ranges between 4 and 29 μm (average 9.01 μm, *n* = 40 cells); the cell breadth is also variable and ranges from 2 to 8 μm (average 4.1 μm, *n* = 40 cells); and the length/breadth ratio (L/B) varies from 1 to 7 (average 2.3, *n* = 40 cells). Consistent with the morphology of other members of Discosea, the locomotive stage of the cell is flat. Cells can show multiple subpseudopodia of variable size that can extend several cell lengths. Subpseudopodia are occasionally furcate. No differentiated uroidal structures were observed, but cells occasionally form trailing filaments. The size of the nuclei ranges between 1 and 2.5 μm (average 1.7, *n* = 20 cells). Some cells were noted to have multiple nuclei (up to five nuclei observed, e.g. Figure 2g and h). Static cells have a slightly shorter length, from 4 to 27 μm (average 7.4, *n* = 40 cells), and slightly wider breadth, from 4 to 13 μm (average 5.2, *n* = 40 cells), in comparison to locomotive cells, which generates a lower L/B ratio with values between 1 and 4 (average 1.4, *n* = 40 cells). Static cells have very variable outlines, with individuals possessing multiple to no subpseudopodia. *A. explorator* possesses a radial-type floating form with several irregular, tapering hyaline pseudopodia, and a cell diameter that varies between 1.5 and 5 μm (average 3.4 μm, *n* = 40 cells) (Fig. 2f, Video S1). In the floating form, we observed contact of subpseudopodia of different cells (Video S2). All measurements above were taken on a 5-day-old culture at 18 °C (cultures were routinely split every 15 d; 5-day-old cultures were selected for measurement because they represented a balance between *A. explorator* cell density versus bacterial overgrowth that would interfere with microscopic measurements). No flagellate stage was observed. No microtubule-organizing center (MTOC) was detected.

The morphology of *A. explorator* cells is distinct from any established order in Centramoebia. In contrast to members of Pellitida, which are characterized by an extremely thick cell coat integrated with the membrane and short subpseudopodia (Smirnov and Kudryavtsev 2005), *A. explorator* possesses only a thin glycocalyx and produces multiple, elongated, and occasionally furcate subpseudopodia. Likewise, the absence of a flexible cuticle or organic microscales, as well as the variability in subpseudopodia length, distinguishes *A. explorator* from members of Himatismenida (Page 1987; Smirnov et al. 2011). Morphological similarity with members of Acanthopodida is more difficult to evaluate because this order is not homogeneous morphologically and mainly supported by molecular phylogeny; although *A. explorator* shares with this order a flattened locomotive stage, the presence of a thin glycocalyx, and sometimes furcate subpseudopodia, these features most likely do not represent synapomorphies and instead may be shared plesiomorphic characters common to all Discosea. However, the lack of MTOC, the length of subpseudopodia (up to several cell lengths) and the presence of multinucleated forms, set *Apostamoeba* apart from all described members of order Acanthopodida (Smirnov et al. 2005; Tice et al. 2016).

*A. explorator* also presents a behavior displaying cytoplasmic bifurcation. For convenience, in this manuscript, we refer to this behavior as the “double-amoeba.” The double-amoeba consists of a bipolarization of a multinucleated cell and the generation of two poles that coexist and act as independent cells for a period of time, which we have always observed to be followed by a final reabsorption (*n* = 10 cells) (Fig. 2c and Video S3). Although the double-amoeba behavior could be found in various cell culture conditions, it could be reliably induced by using low-nutrient medium.

**Figure 3:**
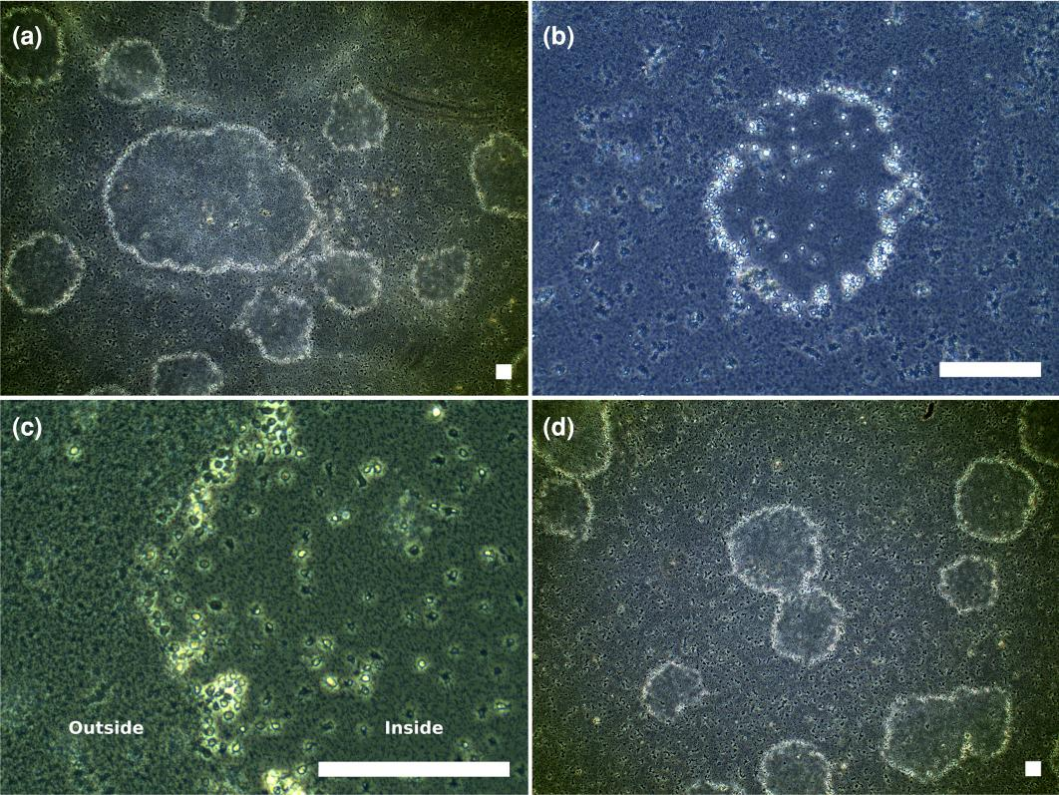
Feeding fronts in *Apostamoeba explorator*. (a) Multiple feeding fronts, generating a ring shape, with sizes ranging from 187 μm to 1625 μm. Also visible are numerous single amoeba cells and prey bacteria. (b) Image of a single feeding front. (c) Close up of a feeding front (the ring is only partially visible in this image). Bacterial density is lower inside versus outside of the ring (see video S4). d) Two merging feeding fronts. All panels are phase contrast microscopy with scale bars = 125 μm.

Lastly, and in addition to the behavior described above, *A. explorator* is able to create feeding fronts (Fig. 3) (Baumgartner et al. 2003). These structures are composed of a front of amoebae advancing together with a fraction of amoebae that remain inside the generated structure, which counterintuitively are the most active ones, in terms of movement (Video S4) (Fig. 3c). The exact conditions that induce this phenomenon have not been found, but we observed that a low number of initial amoeba cells and a pre-established bacterial mat could increase the probability of the generation of these feeding fronts. While other species (mostly isolated from soils) are known to generate fruiting bodies after forming feeding fronts and exhausting the available prey (Smith et al. 2014), we never observed such structures in either aquatic or solid media. It should also be noted that *A. explorator* never exhausted the available prey, as it was cultivated in nutrient-rich media. Therefore, even if *A. explorator* were capable of producing fruiting bodies, we might not have been able to detect them under the conditions we tested.

### Correspondence analysis of clusters of orthologous groups content in Amorphea

A recent study introduced an approach to compare evolutionary trajectories of gene content between major lineages (Ocaña-Pallarès et al. 2022), based on COG content and correspondence analysis (CA). In our study, we extended the *Ocaña-Pallarès* et al. method to analyze Amorphea-wide functional evolution by adding Amoebozoa, a large and diverse closely-related lineage to animals and fungi, and incorporating the transcriptomes of *V. septentrionalis* and *A. explorator* that we sequenced and assembled. Additionally, we performed an ancestral reconstruction in order to recover the COG profile of the Amoebozoa, Fungi, and Metazoa common ancestors, so as to produce a more complete view of the evolution of functional content in Amorphea.

**Figure 4:**
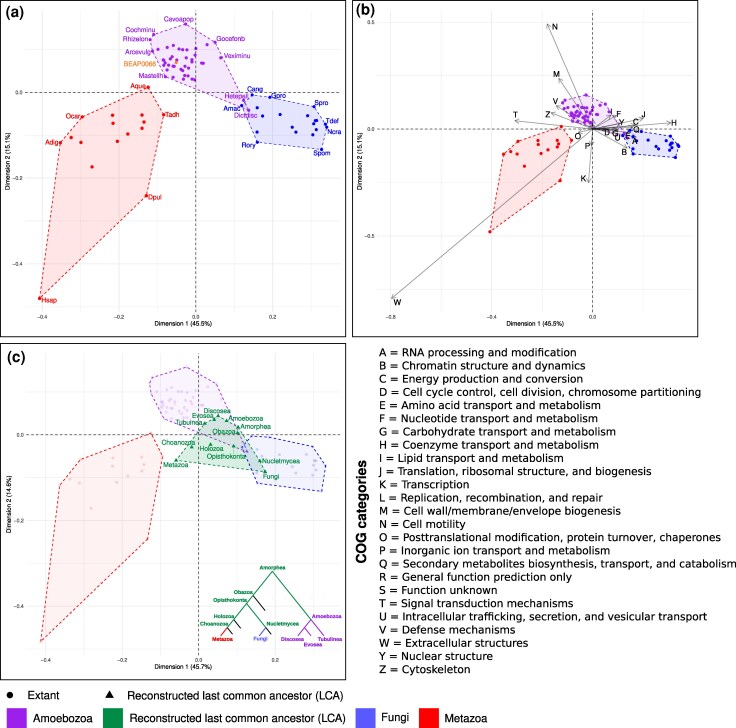
Correspondence analysis (CA) of clusters of orthologous groups (COG) functional category compositions of Amoebozoa, Metazoa, Fungi and their last common ancestors. (a) First two dimensions of CA. Each point represents a single species (genome or a transcriptome; Figure S6b shows that data source does not have a significant impact). Selected points are labeled with species abbreviations: Metazoa (Adig = *Acropora digitifera*, Aque = *Amphimedon queenslandica*, Dpul = *Daphnia pulex*, Hsap = *Homo sapiens*, Ocar = *Oscarella carmela* and Tadh = *Trichoplax adhaerens*), Fungi (Amac = *Allomyces macrogynus*, Cang = *Catenaria anguillulae*, Gpro = *Gonapodya prolifera*, Ncra = *Neurospora crassa*, Rory = *Rhizopus oryzae*, Spom = *Schizosaccharomyces pombe*, Spro = *Sporobolomyces roseus*, and Tdef = *Taphrina deformans*) and Amoebozoa (Arcevulg = *Arcella intermedia*, BEAP0066 = *Apostamoeba explorator*, Cavoapop = *Cavostelium apophysatum*, Cochminu = *Cochliopodium minutoideum*, Dictdisc = *Dictyostelium discoideum*, Gocefonb = *Gocevia fonbrunei*, Hetepall = *Heterostelium pallidum*, Masteilh = *Mastigella eilhardi*, Rhizelon = *Rhizomastix elongata* and Veximinu = *Vexillifera minutissima*). Figure S3 shows the same plot with all points labeled with their corresponding species name. (b) Identical CA to panel A, with individual COG category projections. (c) CA with the addition of last common ancestors (green) inferred by orthologous gene identification (OrthoFinder2) and Wagner parsimony (Notung). The relationship among last common ancestors is indicated in a cartoon tree (inset).

The correspondence analysis shows a clear separation of Amoebozoa, Fungi, and Metazoa into three clusters, indicating that each group exhibits an independently conserved relative composition of COG categories (Fig. 4a, Figure S4). This was also reflected in comparisons between each pair of groups (Figure S5). We note that of the 54 amoebozoans included in the analysis, the two dictyostelid social amoebae *Dictyostelium discoideum* and *Heterostelium pallidum* are positioned more closely to Fungi than to Amoebozoa within the correspondence analysis. Notably, these are two amoebozoans with life histories that include differentiated multicellular stages. The presence of other species inside the Amoebozoa cluster, like *Physarum polycephalum*, with a complex life cycle, yet without differentiated multicellularity, suggests that the atypical placement of *Dictyostelium* and *Heterostelium* may reflect lineage-specific metabolic expansions rather than a general feature of complex life cycles in Amoebozoa. We measured the relative differences (normalized by the larger value) between the COG category content of *D. discoideum* and *H. pallidum* and that of their closest representatives in Amoebozoa (*Acanthamoeba castellanii*) and Fungi (*Allomyces macrogynus*), in terms of their position in the CA (Figure S4), to determine which functional categories draw the dictyostelids toward the Fungi cluster. We also repeated the same analysis using the average values of all Amoebozoa (excluding dictyostelids) and Fungi rather than those of *A. castellanii* and *A. macrogynus*, to avoid potential biases from comparisons to single species. Comparing the two approaches allowed us to identify the COG categories that were consistently closer in value to each supergroup. The differences in COG profile positions of the dictyostelids were larger relative to Amoebozoa in both analyses, indicating that the dictyostelid profile is generally more similar to Fungi. Specifically, the content of categories Q (secondary metabolites biosynthesis, transport, and catabolism) and A (RNA processing and modification) in dictyostelids was consistently more similar to Fungi than to Amoebozoa, whereas categories C (energy production and conversion), D (cell cycle control, cell division, chromosome partitioning), E (amino acid transport and metabolism), and Z (cytoskeleton) showed values more similar to other Amoebozoa (File S1).

Different COG categories appear to be associated with clusters for different lineages in Amorphea: the Amoebozoa cluster is associated with a higher proportion of the M and N COG categories (cell wall/membrane/envelope biogenesis and cell motility), whereas the fungal gene repertoire is more associated with COGs with functions in metabolism (C, E, F, G, H, I, and Q among others) and Metazoa presents a very strong association with the W COG category (extracellular structures) and with categories T and K (transcription and signal transduction) (Fig. 4b, Figure S5b, Figure S6a). Inside Amoebozoa, there is no clear separation among the Discosea, Tubulinea, and Evosea lineages; their divergence might not be reflected in COG categories and therefore would not be detected with our methodology (Figure S7), although we observed hints of conserved evolution of gene repertoire at lower taxonomic levels (at the taxonomic level of orders) (Berney et al. 2017) (Figure S8).

In our ancestral reconstruction (Fig. 4c), the Amorphea last common ancestor (LCA) is located between the Amoebozoa and Fungi clusters. The Amoebozoa LCA is very similar to the Amorphea LCA, retaining a higher content of M and N categories (cell membrane biogenesis and cell motility) than Metazoa and Fungi LCAs, which underwent a decrease in these categories (Figure S9a). The Metazoa LCA falls outside the cluster of extant metazoan species, supporting convergent genomic trajectories following their separation from the metazoan LCA; most prominently, multiple animals appear to have independently gained genes annotated in the W category (see Discussion). Within Amoebozoa, the Tubulinea LCA appears to be more divergent from the Amoebozoa LCA than the Evosea or Discosea LCAs (Fig. 4c, Figure S9b), which could be biased by the fact that the amoebozoan classes are not equivalent in terms of phylogenetic divergence, as they were created by morphology-biased protistologists and, from a phylogenetic point of view, Tubulinea is the equivalent of orders inside Discosea.

We performed several *in silico* controls in order to validate the integrity of our results. The *Ocaña-Pallarès et al.* approach used only proteomes derived from genomic data, but in order to obtain sufficient Amoebozoa sampling we also used proteomes derived from transcriptomes. Transcriptomes may be more incomplete and prone to errors than genomes; nevertheless, we found that, for the subset of species that had both genomes and transcriptomes available, the resulting clusters in the correspondence analysis were not significantly different between the two datasets, with the transcriptome gene repertoire for some species actually clustering more tightly with other species in the same lineage than the corresponding genome (Figure S6). Next, ancestral reconstruction results could be a reflection of the inherent bias of the method used for reconstruction (Gàlvez-Morante et al. 2024). However, we found consistent results with two different ancestral reconstruction methods, Wagner parsimony, and maximum likelihood (Figure S10) (K. Chen et al. 2000; Guéguen et al. 2013). Finally, we provided evidence that the clustering of Amoebozoa is driven by a biological signature and not simply by the fact that they are neither Fungi nor Metazoa, after introducing artificial species with random COG composition that grouped completely outside of the biological clusters (Figure S11).

### Identification of overrepresented Pfam domain clans

Our results from the correspondence analysis of COG content in Amorphea shed light into the evolution of the studied supergroups. However, the limited number of COG categories makes it challenging to generate precise biological interpretations. In this context, we decided to perform a second complementary analysis based on Pfam families, as they represent structural, evolutionary, and functional information. We focused on Pfam families grouped together at the clan level to further classify this information by function. Pfam clans are groups of related Pfam families (also known as Pfam domains) sharing evolutionary, structural, or functional relationships. They address limitations of sequence-based Pfam family classifications by integrating deeper evolutionary connections and structural similarities that might not be evident through sequence alone (Finn et al. 2006). At the same time, the usage of Pfam clans instead of Pfam families is a better representation of function as Pfam families often split proteins that perform the same function into separate groups, obscuring functional relationships between proteins that have evolved differently but still carry out the same role.

**Figure 5:**
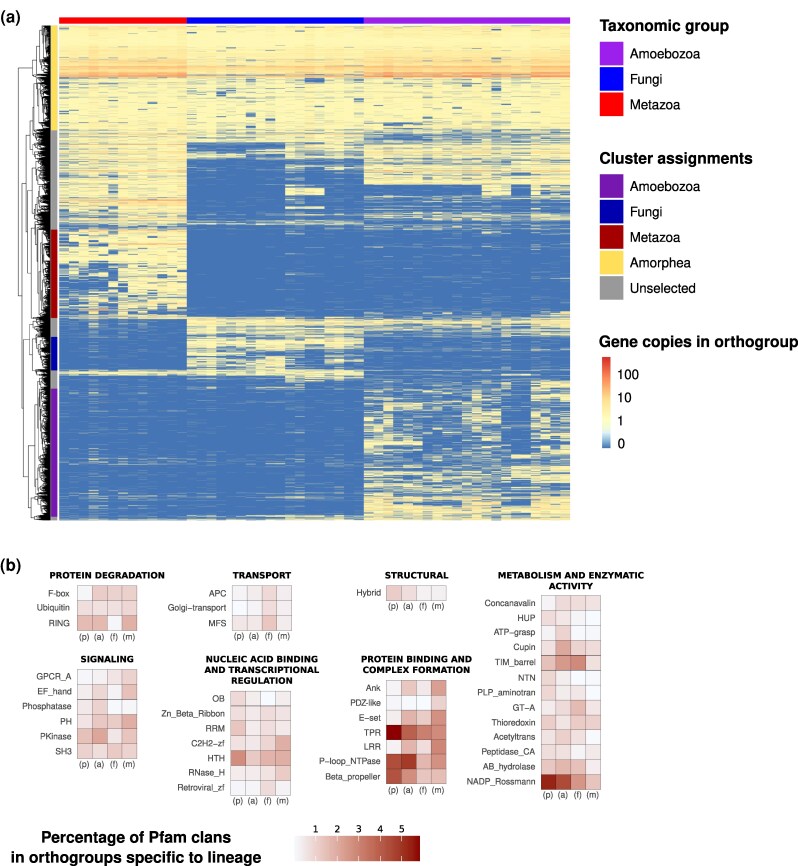
Evolution of orthologous gene families and Pfam clans in Amorphea. (a). Clustering analysis (vertical axis) of gene counts for orthogroups identified in species with genomes or transcriptomes of high completeness (BUSCO eukaryota_odb10 > 80%, horizontal axis). Gene counts were log10 normalized. Colored vertical bars to the left of the heatmap indicate manual assignments of hierarchical clusters as being specific to a taxonomic lineage. (b) Relative proportions (expressed as percentages) of Pfam clans present in orthologous gene families assigned to specific lineages (p = Amorphea, a = Amoebozoa, f = Fungi, and m = Metazoa). Rows represent percentages of Pfam clans present in all gene families assigned to a lineage, for all Pfam clans present in the top 20 most abundant in any of the four lineages. Pfam clans are grouped by primary/most frequent function. The Pfam domain Mito_carr is not assigned to a clan and is listed by its domain name.

We performed an orthology inference for all proteins in the studied extant species with OrthoFinder2 and hierarchically clustered orthogroups by the number of representative genes present in each species. Next, we automatically defined clusters and selected groups of orthogroups specific to each lineage by cutting the dendrogram, in order to generate phylogenetically coherent orthogroup clusters. Finally, we translated orthogroup presence within each detected cluster into Pfam clan content, allowing us to determine the relative abundance of Pfam clans (Fig. 5b).

We observed a cluster of orthologous gene families present in all analyzed species, and clear clusters of genes specific to Amoebozoa, Fungi, or Metazoa (highlighted in different colors on the vertical axis of Fig. 5a). In addition to clusters of orthologs estimated to be exclusive to these groups, we also observe shared clusters between taxonomic groups. There are very few genes in clusters exclusive to Fungi and either Metazoa or Amoebozoa, but there is a clear set of genes largely present in Metazoa and Amoebozoa, while absent in Fungi, which is evidence that these genes may have been lost in Fungi after their divergence from animals.

We found the following Pfam clans to be overrepresented specifically in the common Amorphea cluster (see Methods for how overrepresentation was calculated): Beta_propeller and TPR (whose primary functions include protein binding and complex formation); HTH, OB, and RRM (nucleic acid binding and transcriptional regulation); HUP, NTN, and PLP_aminotran (metabolism and enzymatic activity); and hybrid (structural). The overrepresented Pfam clans in the Amoebozoa-specific cluster were PKinase and phosphatase (Signaling); and ATP-grasp, acetyltrans, and cupin (metabolism and enzymatic activity). In Fungi, GT-A (metabolism and enzymatic activity); Retroviral-zf (nucleic acid binding and transcriptional regulation); APC, MFS, and Golgi-transport (Transport) were overrepresented. And, in Metazoa, LRR, E-set, Ank, and PDZ-like (protein binding and complex formation); EF hand, PH, and GPCR_A (Signaling); and C2H2-zf and RNase_H (nucleic acid binding and transcriptional regulation) (File S2).

## Discussion

In this study, we explored the evolution of function across the three major amorphean lineages, Amoebozoa, Fungi, and Metazoa, through the combination of COG categories and correspondence analysis. Based on previous work that revealed contrasting evolutionary trajectories in Fungi and Metazoa, we extended this methodology to include Amoebozoa, revealing that it exhibits a third independent evolutionary trajectory. The inclusion of two new amoebozoan isolates, *V. septentrionalis* and *A*. *explorator*, together with the characterization of the latter, further expanded our phylogenetic and functional sampling, providing valuable insight into the evolution of Amoebozoa.

*A. explorator* has a very interesting phylogenetic position and represents a new amoebozoan lineage that has been hidden in environmental samples until its characterization by light and electron microscopy, coupled with transcriptome sequencing and phylogenomics. This newly discovered lineage, with strong phylogenetic support, includes numerous environmental sequences representing diversity outside currently described amoebozoan lineages (Figure S2). Our recovered phylogenomic topology (Fig. 1) shows Tubulinea as sister to Evosea + Discosea, consistent with the results of Tekle et al. (Hess et al. 2019; Tekle et al. 2022), and in contrast to Kang et al. (Kang et al. 2017), which placed Discosea as sister to Tubulinea + Evosea. This disagreement reflects a long-standing debate, likely driven by differences in gene and taxon sampling, as well as phylogenetic models. The placement of Tubulinea as sister to Evosea + Discosea is present in our phylogenomic reconstruction with strong statistical support, adding independent evidence in favor of this topology.

The intriguing behaviors of *A. explorator* that we observed could also contribute to a better understanding of Amoebozoa cell biology. First, we hypothesize that it could be possible that the double-amoeba behavior results when an amoeba becomes multinucleated and the different nuclei in the cell act as local translation hubs, providing contradicting orders to the local cytoskeleton, which eventually leads to different parts of the cell moving in different directions in an uncoordinated manner. This behavior could be a way to explore more terrain in conditions of low food availability. There are similar behaviors in the genera *Flamella* (Glotova and Smirnov 2017), which generate plasmodia that end up dividing, and *Flabellula* (Fenchel 2010), which are able to create a resting stage characterized by flat multinucleated cells that can rapidly multiply in certain conditions (the so-called “Salvador Dalí” morphotype), but the closest behavior is found in Vampyrellida (Berney et al. 2013), which extend cytoplasmatic arms to hunt for prey, and especially the behavior described for the expanded morphotype of *Leptophrys vorax* (Hess et al. 2012), which generates cells that can be drawn out to considerable length appearing as two cell bodies only connected via a thin cytoplasmic strand. Other similar behaviors exist in *Dictyostelium* (Taira and Yumura 2017) and *Entamoeba* (Biron et al. 2001), both caused by incomplete cell divisions and related to the need for attachment (*Dictyostelium*) or intra-specific cooperation for a successful division (*Entamoeba* “Midwives”), but we do not believe this is the case for *A. explorator* as the “double-amoeba” was never observed to divide and the culture is capable of typical cell division. Second, in the case of the feeding fronts, we believe that the generation of such structures improves the efficiency of preying on bacterial mats (Baumgartner et al. 2003). Collective feeding strategies are widespread among amoebae, including taxa outside Amoebozoa, such as *Euhyperamoeba fallax* (Heterolobosea), which forms aggregates and plasmodium-like structures that enhance predation efficiency (Seravin and Goodkov 1987). Third, the contact of pseudopodia that we observed in the floating form is superficially similar to tunneling nanotubes, which are cell bridges that mediate the intercellular transfer of organelles, plasma membrane components and cytoplasmic molecules, which have been studied mainly in Metazoa (Gerdes and Carvalho 2008), although additional investigation on the nature of these pseudopodial contacts (ultrastructure, molecular components) is required to determine whether the two structures could be homologous, or whether our observations instead represent occasional contact of pseudopodia in a dense culture of floating forms.

In our analysis of gene repertoire composition, Amoebozoa, Metazoa, and Fungi showed three distinct evolutionary trajectories. Initially, we calculated the relative composition of COG categories in proteomes of extant species in Amoebozoa, Metazoa, and Fungi. Next, we used ancestral reconstruction to estimate the COG profiles of their Amorphea ancestors. These data enabled us to perform correspondence analysis to explore differences in COG composition based on the taxonomy of extant and reconstructed ancestral species. Our findings align with previous studies (Ocaña-Pallarès et al. 2022), replicating associations with COGs related to animal multicellularity in the case of Metazoa and COGs related to metabolism in the case of Fungi. In contrast, the addition of new data in our study reveals that Amoebozoa display a third evolutionary trajectory enriched in the M (cell wall/membrane biogenesis) and N (cell motility) COG categories, likely to be related to the amoeboid morphologic flexibility, crucial for motility, phagocytosis, and generation of various life stages and structures. It is very interesting to observe that dictyostelids cluster closer to Fungi than to Amoebozoa, while other amoebozoans with complex life cycles like *Physarum polycephalum* cluster with the rest of Amoebozoa and are not pulled toward Fungi. This observation suggests that specific evolutionary changes in dictyostelids, that are not necessarily generalizable to all Eumycetozoa, may have led to the observed shift in their COG profiles, although further research is needed to confirm this hypothesis. The reconstruction of COG profiles of Amorphea ancestors revealed intriguing insights into evolutionary relationships. Our findings suggest that the ancestral Amoebozoa proteome is closer than the last common ancestor of Metazoa or Fungi to the last common ancestor of Amorphea, with relatively few changes in a period of evolutionary stasis, in terms of the distribution of genomic functions among COG categories. In contrast, metazoan extant species exhibit a high degree of difference from the Metazoa last common ancestor, potentially reflecting convergent evolution during the birth of the different lineages within Metazoa (although it is also possible that genes from different lineages were artificially separated during the process of orthogroup inference, which would have caused them to be absent in the metazoan LCA).

To add to our conclusions based on Amorphea relative COG content, we decided to use Pfam family clans in a complementary analysis. COG categories are a useful tool that give us a general overview on functional content, but Pfam clans offer more specific functional detail than COGs. Pfam clans are more suited to our study than plain Pfam families (also known as domains), as Pfam clans group together families of proteins that perform the same function. We gathered all proteins in the studied extant species, inferred orthologous gene families and hierarchically clustered them. We detected putative Amoebozoa-specific, Fungi-specific and Metazoa-specific clusters, as well as a cluster of orthologous gene families present in all extant species analyzed, and several clusters with more complex evolutionary histories. Next, we translated protein presence within each cluster of orthologous gene families into Pfam clan content, allowing us to determine the relative abundance of Pfam clans. In the case of Metazoa, we can again detect a specialization toward animal multicellularity with Pfam clans like LRR or E-set, which are often found in genes with functions in cell–cell adhesion and the animal immune system, and PDZ-like, which is critical for the assembly of supramolecular complexes and signal transduction (Sheng and Sala 2001; Jeleń et al. 2003). Fungi showed a higher specialization toward Pfam clans related to transport, such as MFS, APC, and Golgi-transport, which could be essential for the uptake and secretion of enzymes, nutrients, and metabolites, as characteristic for a fungal lifestyle. The detection of a large shared cluster between Amoebozoa and Metazoa, together with the fact that the Fungi-specific cluster is clearly smaller than the others, are consistent with observations that gene loss played a role in fungal evolution (Merényi et al. 2023). In Amoebozoa, we could detect a specialization in phosphate-mediated signal transduction, with a diversification of protein kinases and phosphatases, which together with the high content in ATP-grasp could be related to the need for a rapid response to diverse environmental stimuli and an expansion related to signal transduction pathways (Lue et al. 2006; Fawaz et al. 2011; Kuhn et al. 2024). Given the high ATP cost of actin filament assembly, this specialization in phosphate metabolism could facilitate fast ATP processing, which could be crucial for rapid pseudopodial development.

This project identified distinct evolutionary trajectories within Fungi, Metazoa, and Amoebozoa by analyzing patterns in COG categories and Pfam family clans. These findings support the hypothesis that the phylogenetic relationships among the lineages are reflected in the relative compositions of their gene repertoires. We hypothesize that the evolutionary trajectory of Amoebozoa, shaped by the detected COG categories and Pfam clans, retained a partially ancestral eukaryotic state optimized for their particular predatory lifestyle. This state is centered on phagocytosis, motility, cellular plasticity, and the capacity to generate rapid responses to environmental stimuli. This specialization toward phagocytosis could have enabled access to bacterial mats, the primary food source in the mid-Proterozoic biosphere, the era in which Amoebozoa first emerged (Tekle et al. 2022).

For future studies, we propose example follow-up experiments to test our hypotheses. A transcriptomic analysis of selected extant Amoebozoa species under starvation conditions, followed by the addition of prey to induce phagocytosis (accompanied by matched no-prey controls) could be performed in order to detect genes upregulated in amoebozoan predation. We could then measure the relative proportions of these genes (if present) in Fungi, Metazoa, Amoebozoa and the reconstructed Amorphea ancestor, to confirm that the genes that Amoebozoa retained from the last common ancestor of Amorphea are indeed overrepresented in predation. The experiment would need to be performed with appropriately matched non-amoebozoan control species that would let us extract Amoebozoa-specific conclusions. Thus, it would be conducted in parallel with cultures of model fungi and experimentally tractable animals, to determine whether the genes upregulated in prey capture are not the same as those in Amoebozoa. A second potential approach would be to knock out ATP metabolism genes found in Amoebozoa and absent in other groups in one or more amoebozoan representatives to observe the effects on pseudopodial formation and locomotion. Knock out experiments of other ATP metabolism genes found across Amorphea (i.e. not exclusive to Amoebozoa) would be a necessary control to elucidate the role of amoebozoan-specific ATP metabolism innovations. As knock out experiments require reliable genetic tools, *Dictyostelium discoideum* would be one appropriate choice of model system.

Overall, in this work, by studying and identifying a broad range of functional evolutionary trajectories within Amorphea, we aimed to deepen our understanding of the molecular and functional innovations underlying eukaryotic cell evolution, with the hope that this knowledge could provide insights into the diversity and complexity of life in eukaryotes.

## Taxonomic summary

### Eukaryota, Amorphea, Amoebozoa, Discosea, Centramoebia

**Superorder Acapostellia Gàlvez-Morante, Berney et Richter, n. superord.**

**Diagnosis:** The most inclusive clade that includes the genera *Acanthamoeba*, *Pellita*, and *Apostamoeba*, but excludes *Cochliopodium*.

**Etymology:** A compound word composed of the three orders which currently compose this superorder: “Aca” from Acanthopodida, “apost” from Apostamoebida, and “ell” from Pellitida.

**Order Apostamoebida Gàlvez-Morante, Berney et Richter, n. ord.**

**Diagnosis:** Amoebae with a flat locomotive stage with multiple, variably-sized subpseudopodia that can extend several cell lengths. Cells can be multinucleated. Radial-type floating form with tapering hyaline pseudopodia. No clear differentiated uroidal structures. No flagellated stage. No microtubule-organizing center is present.

**Etymology:** Named after the Greek term “Apostolos,” meaning “the one who is sent away” or “messenger,” in reference to its high 18S rRNA gene phylogenetic divergence.

**Family Apostamoebidae Gàlvez-Morante, Berney et Richter, n. fam.**

**Diagnosis:** With the characteristics of the order Apostamoebida.

**Etymology:** Named after the Greek term “Apostolos,” meaning “the one who is sent away” or “messenger,” in reference to its high 18S rRNA gene phylogenetic divergence.

**Type genus:**  *Apostamoeba*.

**Zoobank registration:** Described under the Zoological Code; ZooBank registration: urn:lsid:zoobank.org:act:B11D7643-565D-43C3-B5B5-7AAE6EFF8E52

**Genus *Apostamoeba* Gàlvez-Morante, Berney et Richter, n. gen.**

**Diagnosis:** Amoebae with a flat locomotive stage with multiple, variably-sized subpseudopodia that can extend several cell lengths. Locomotive stage cell outline is highly variable, delimited by a thin glycocalyx. Cells can be multinucleated. Radial-type floating form with several irregular, tapering hyaline pseudopodia. No clear differentiated uroidal structures, but occasionally forms trailing filaments. Static cells have very variable outlines, with individuals possessing multiple to no subpseudopodia. It frequently exhibits a “double-amoeba” behavior under low nutrient conditions, where a multinucleated cell temporarily splits into two independent poles before reuniting, and has the capacity to form feeding fronts to feed on bacterial mats. It does not possess a flagellated stage and no microtubule-organizing center has been detected.

**Etymology:** Named after the Greek term “Apostolos,” meaning “the one who is sent away” or “messenger,” in reference to its high 18S rRNA gene phylogenetic divergence and the fact that it is the first described genus of an undiscovered vast amount of environmental diversity inside Apostamoebida. Apostamoeba is feminine.

**Type species:**  *Apostamoeba explorator*.

**Zoobank registration:** Described under the Zoological Code; ZooBank registration: urn:lsid:zoobank.org:act:86CE928D-D725-440D-9430-64A50CDA6A4C

**Species *Apostamoeba explorator* Gàlvez-Morante, Berney et Richter, n. sp.**

**Diagnosis:** Locomotive cells, excluding subpseudopodia, range from 4 to 29 μm long (average 9.01 μm) and 2 to 8 μm wide (average 4.1 μm), producing a length-to-breadth ratio of 1 to 7 (average 2.3), while their nuclei measure 1 to 2.5 μm (average 1.7 μm). Static cells have a slightly shorter length range, from 4 to 27 μm (average 7.4), and slightly wider breadth range, from 4 to 13 μm (average 5.2), in comparison to locomotive cells, which generates a lower L/B ratio with values between 1 and 4 (average 1.4). Floating form with a diameter of 1.5 to 5 μm (average 3.4 μm).

**Etymology:** The latin term “explorator” refers to its ability and behaviors to investigate its surroundings.

**Type locality:** Subsurface water, Mediterranean Sea, Blanes Bay, Spain, 41°40′ N/2°48′ E.

**Type material:** The name-bearing type (an hapantotype) is a culture containing *Apostamoeba explorator* (strain BEAP0066) and mixed bacterial species deposited in the Culture Collection of Algae and Protozoa (CCAP) with accession number CCAP 2503/1.

**Gene sequence:** The rRNA gene sequence (18S, ITS1, 5.8S, ITS2, 28S) of strain BEAP0066 is deposited in Genbank as PV590302.

**Zoobank registration:** Described under the Zoological Code; ZooBank registration: urn:lsid:zoobank.org:act:17F3F7DB-0744-40F0-984B-85D8EB6EF141

This publication (work) is registered in ZooBank as: urn:lsid:zoobank.org:pub:6250E286-180E-410D-BD8B-C08EA97D579F

## Methods

### Isolation and culture

*Apostamoeba explorator* was isolated from the coast of Spain, in Blanes Bay (41°40′ N/2°48′ E) (salinity = 37 ‰) in November 2021 through the dilution of surface marine water in RS medium (1 nutrient medium [NM]: 10 non-nutrient medium [NNM] concentration) (salinity = 33 ‰). RS medium is a fully defined medium designed to grow a wide range of marine protists. It is composed of two components: the NM and the NNM (https://mediadive.dsmz.de/medium/P4, https://mediadive.dsmz.de/medium/P5).

*A. explorator* was maintained in 25 cm^2^ flasks with a total volume of 10 mL, with a split frequency of 1 split per 1.5 weeks at 18 °C, in the presence of light. The splits were performed by adding 9 mL of R/S medium (1 NM: 10 NNM concentration, also viable at 1:50 and 1:100) (salinity = 33 ‰) and 1 mL of the previously growing culture, with scraping.

*Vannella septentrionalis* (strain BEAP0079) was isolated from the coast of Spain, in Blanes Bay (41°40′ N/2°48′ E) (salinity = 37 ‰) in 2021 through the dilution of surface marine water in RS medium (1 NM: 10 NNM concentration) (salinity = 33 ‰). *V. septentrionalis* was maintained following the same strategy described above, but at a temperature of 12 °C, in the absence of light and at RS concentration 1 NM: 50 NNM.

### Light microscopy

For differential interference contrast microscopy, cells were seeded on a glass-bottom dish. Imaging was conducted using a Zeiss Axio Observer 7 inverted microscope equipped with a 63× oil-immersion lens. Digital images were processed using Fiji software (ImageJ 1.54f) (Schindelin et al. 2012); when the size of a cellular feature (e.g. the nucleus) was measured, the focal plane containing its maximal extent was selected for measurement. For Phase Contrast microscopy cells were seeded in six-well plates (CULTEK, diameter 3.5 cm). Cells were observed using a Zeiss Axio Observer 7 inverted microscope and pictures were taken with a mobile phone (OnePlus Nord 2 5G), through an adapter (SOLOMARK phone adapter, ASIN: B0BC7PC974, Manufacturer reference: S-optics-A0058).

For morphological measurements, cells were seeded in glass-bottom wells (IBIDI µ-Slide 8 Well high) at room temperature 30 to 60 min prior to observation, and a single measurement was taken from each individual locomotive cell. Locomotive cells were obtained by seeding a 100 μL of 5-day-old culture into 300 μL of RS medium.

### Scanning electron microscopy

Cells were fixed with 2.5% glutaraldehyde for 3 h at room temperature and then seeded on membranes with 0.8 μm pores (WHA10417301, MERCK Chemicals and Life Science) via filtering. The samples were dehydrated through a graded ethanol series and dried by critical point with liquid carbon dioxide in a Leica EM CPD300 unit (Leica Microsystems, Austria). The dried filters were mounted on stubs with colloidal silver and then were sputter-coated with gold in a Q150R S (Quorum Technologies, Ltd.) and observed with a Hitachi SU8600 field emission scanning electron microscope (Hitachi High Technologies Co., Ltd., Japan) in the Electron Microscopy Service of the Institute of Marine Science (ICM-CSIC), Barcelona.

### Transmission electron microscopy

Sample preparation was performed in the Electron Cryomicroscopy Unit at the Scientific and Technical Centers of the University of Barcelona. Samples were concentrated by centrifugation (2,000×g, 5 min) and resuspended in 20% BSA in artificial sea water. Most of the supernatant was removed and the concentrated cells cryo-immobilized using a Leica HPM100 high-pressure freezer (Leica Microsystems, Vienna, Austria). Samples were freeze-substituted in pure acetone containing 2% (w/v) osmium tetroxide and 0.1% (w/v) uranyl acetate at −90 °C for 72 h in an EM AFS2 (Leica Microsystems, Vienna, Austria). Later, they were warmed up to 4 °C at a 5 °C/h slope, kept at 4 °C for 2 h, and transferred to room temperature and kept for 2 h in darkness. Samples were washed in acetone at room temperature, infiltrated in increasing concentrations of Epon-812 resin in acetone until purely Epon-812. Then, they were embedded and polymerized in Epon-812 at 60 °C for 48 h. Ultrathin sections of 60 nm were obtained with a UC6 ultramicrotome (Leica Microsystems, Vienna, Austria) and placed on Formvar-coated copper grids. Sample sections were stained with 2% (w/v) uranyl acetate for 30 min, lead citrate for 5 min and examined in a TEM Jeol JEM 1010 (Gatan, Japan) equipped with a tungsten cathode. Images were acquired at 80 kV with a 1 k × 1 k CCD Megaview III camera.

### Immunofluorescence microscopy

Cells were seeded on coverslips pre-treated with poly-L-lysine to enhance cell adherence to the glass surface, washed once with artificial sea water (ASW), and fixed with a solution of 4% formaldehyde in ASW for 5 min. Cells were washed for 5 min with ASW, blocked and permeabilized in a solution containing 1% BSA and 0.3% Triton X-100 in ASW for 1 h. After, cells were incubated with 132 nM phalloidin 488 (A12379, ThermoFisher Scientific) in ASW at room temperature for 30 min, in order to stain actin. DNA was stained with 0.2 μM Draq-5 (62251, ThermoFisher Scientific) for 15 min. Coverslips were mounted in ProLong Gold antifade reagent (P36934, Thermo Fisher Scientific).

The observation of the samples was carried out at the Advanced Light Microscopy Unit located at the Centre for Genomic Regulation (CRG) in Barcelona, using a Zeiss LSM 980 confocal microscope equipped with Airyscan 2 super-resolution (405, 488, 561, and 639 nm lasers) and a 63 × lens. Digital images were acquired in Z-stacks spanning the full cell volume and later processed using Fiji software (Schindelin et al. 2012).

### 18S rRNA gene sequencing

A sample of 50 mL (two 75 cm^2^ flasks with a total volume of 25 mL) was used for 18S cloning. The cells were centrifuged for 20 min at 13,000×g and 4 °C. DNA was extracted using the DNeasy PowerSoil Pro kit (QIAGEN) and the 18S rRNA gene was amplified, in the case of *Apostamoeba explorator*, by PCR using universal eukaryotic primers 42F-1747R (CTCAARGAYTAAGCCATGCA - CCTTCYGCAGGTTCACCTAC) and, in the case of BEAP0079, by gradient PCR (from 47 °C to 58 °C, optimal temperature = 52 °C) using a vannelid-specific forward primer together with a universal eukaryotic reverse primer (CCATGCAAGTCTAAGTATAAATCAT—CCTTCYGCAGGTTCACCTAC) (López-García et al. 2003; Vaulot et al. 2022). Amplification products were purified using NZYGelpure kit (NZYTech), cloned using the TOPO-TA Cloning kit (Invitrogen) and transformed into *E. coli* cells following LacZα-complementation. Positive clones were selected and amplified by PCR with vector-specific primers M13F-M13R (GTAAAACGACGGCCAGT - CAGGAAACAGCTATGAC). Sequencing was performed by Eurofins genomics using Sanger sequencing. The resulting sequences were base called using phred (Ewing et al. 1998) with the parameters “-trim_alt” “-trim_cutoff 0.01” and assembled with phrap (de la Bastide and McCombie 2007) with the parameter “-repeat_stringency 0.4” and the consensus sequence was exported with consed (Gordon and Green 2013). Assembled sequences were identified through BLAST versus EukRibo (Berney et al. 2022).

While the 18S sequences obtained with these methodologies were used to initially identify the two species, for 18S phylogenetic trees and upload to NCBI, we instead used the more complete sequences assembled by phyloFlash (Gruber-Vodicka et al. 2020) for BEAP0079 and Trinity (Grabherr et al. 2011) for *A. explorator* (contig TRINITY_DN167_c0_g1_i10, which included the full rRNA operon).

### RNA extraction

In order to extract and sequence total RNA we used a similar experimental approach as the one described by Richter and colleagues (Richter et al. 2018). Prior to RNA isolation, both strains were grown in large batches using two 75 cm^2^ Rectangular Canted Neck Cell Culture Flasks with Vented Caps (353136, Corning Life Sciences), each containing 50 ml of medium.

We isolated total RNA from both strains by using the RNAqueous kit (AM1912, ThermoFisher Scientific). RNA concentration was measured using a NanoDrop One/One C UV-Vis Microvolume Spectrophotometer (ThermoFisher Scientific). We digested genomic DNA using the TURBO DNA-free kit (AM1907, ThermoFisher Scientific) according to the manufacturer's instructions and removed DNase using DNase Inactivation Reagent. The quality evaluation of extracted total RNA, the library preparation, and the sequencing were carried out at the CRG Genomics Core Facility in Barcelona. The quality of extracted total RNA was assessed by Bioanalyzer 2100 RNA Pico chips (Agilent Technologies). To prepare stranded libraries, the NEBNext Ultra II Directional RNA Library Prep Kit Total RNA was used, applying double the normal rounds of PolyA selection prior to cDNA synthesis to reduce the excess of bacterial RNA. Finally, the RNA was sequenced by using Illumina NextSeq 2000 (2 × 150 bp).

### *De novo* transcriptome assembly and proteome generation

The quality assessment of the sequenced RNA was performed using FastQC v0.11.9 (Andrews, 2010), while evaluating the purity of the sequences using phyloFlash v3.4 (Gruber-Vodicka et al. 2020), a tool designed to assess the 16S/18S rRNA gene taxonomic composition of metagenomic datasets. The elimination of artifacts and adapter sequences was achieved via fastp v0.23.2 (S. Chen et al. 2018) with the following parameters: --low_complexity_filter, --cut_front, --cut_tail, --cut_right, --cut_front_window_size 1 --cut_tail_window_size 1, --cut_right_window_size 4, --cut_mean_quality 5, --trim_front1 12 --trim_front2 12, --trim_poly_g, --trim_poly_x --adapter_sequence = AGATCGGAAGAGCACACGTCTGAACTCCAGTCA –adapter_sequence_r2 = AGATCGGAAGAGCGTCGTGTAGGGAAAGAGTGT. *De novo* transcriptome assembly was performed using Trinity v2.14.0 (Grabherr et al. 2011) with parameter –SS_lib_type RF.

We decontaminated the newly assembled transcriptome by blastn against a set of potential contaminant genomes (species with a 16S/18S detected by phyloFlash or Trinity; File S3) and removing matching contigs with a percentage identity of ≥98% and match length >100. WinstonCleaner with default parameters was also used to remove cross-contamination between samples sequenced in the same run (https://github.com/kolecko007/WinstonCleaner). The FCS-adaptor v.0.5.5 tool was also run to further decontaminate our transcriptomes, which cleaned two contigs from *A. explorator* and three contigs from BEAP0079 (Astashyn et al. 2024).

Subsequently, protein prediction was performed using TransDecoder v5.5.0 with the “-S” option for strand specificity (https://github.com/TransDecoder/TransDecoder) and Pfam protein annotation was carried out with InterProScan 5.56-89.0 with default parameters except for –disable-precalc and –applications Pfam, Phobius (Blum et al. 2021). To examine the completeness of our transcriptomes, we searched for the presence of a set of conserved, single-copy orthologous genes by employing BUSCO v5.3.2 with the Eukaryota odb10 dataset (Simão et al. 2015).

### Phylogenetic analyses and genetic diversity related to *Apostamoeba*

An alignment of the full 18S sequences of representatives of the lineage diversity within class Discosea was constructed in BioEdit version 7.2.5 (Hall 1999) and manually optimized using a published secondary structure model (Wuyts et al. 2000) that was also used for the selection of unambiguously aligned positions. For taxa with transcriptome or genome data available in EukProt (Richter et al. 2022), a complete 18S rRNA sequence was extracted from the assembled transcriptome contigs or genome shotgun sequences and was used to replace the corresponding GenBank sequence to ensure the best possible 18S rRNA coverage and sequence quality. We also included environmental clones from a long-read environmental survey of various habitat types (Jamy et al. 2020) that represent interesting undescribed diversity within Amoebozoa (see File S4 for a list of all 18S rRNA sequences used in our analyses). Slow-evolving members of classes Tubulinea and Variosea were used as outgroups. Some divergent lineages inside Discosea were excluded to minimize tree reconstruction artifacts. This exclusion mostly concerned individual genera within orders that have other representatives in the dataset, such as *Vexillifera* inside Dactylopodida, *Microglomus* inside Mycamoebida, *Thecamoeba* inside Thecamoebida, or *Ripella* inside Vannellida. However, order Himatismenida was entirely excluded from the analysis because it artifactually clustered with Tubulinea instead of as sister to other Centramoebia (data not shown). A maximum likelihood phylogenetic tree was inferred using RAxML version 8.2.10 (Stamatakis 2014) from this 18S dataset of 100 species and 1545 nucleotide positions (Files S5–S7). We used the GTRGAMMA model of evolution with automatic bootstrap convergence (autoMRE), which stopped after 300 replicates.

In addition, the environmental rRNA diversity related to *Apostamoeba* was investigated using the EukBank database (Berney et al. 2017, 2023) by a blastn search using the V4 region of the 18S sequence of isolate BEAP0066 as a sequence query. A total of 62 V4 ASVs related to *Apostamoeba* were identified in that way. To check that all retrieved sequences are truly more closely related to isolate BEAP0066 than to any other amoebozoan and to visualize that V4 diversity, the ASVs were aligned manually to the V4 region extracted from isolate BEAP0066 and a selection of other members of class Discosea, as described above for the full 18S sequence dataset. In this case, members of Himatismenida could be included. Ambiguously aligned positions were removed and the remaining 330 nucleotide positions were used to build a maximum likelihood phylogenetic tree with RAxML as described above (Figure S2, Files S4, S8–S10), but with 1000 rapid bootstrap inferences.

The predicted proteome of *A. explorator*, together with an increased sampling for Amoebozoa (File S11), underwent PhyloFisher's protocol for phylogenomic assembly (Tice et al. 2021), following the standard specified instructions (https://thebrownlab.github.io/phylofisher-pages/). The PhyloFisher protocol includes the collection of homologs for the input species with HMMER (Mistry et al. 2013); construction of gene trees: aligned using MAFFT (Katoh and Standley 2013), filtered with PREQUAL (Whelan et al. 2018) + DIVVIER (Ali et al. 2019), trimmed with BMGE (Criscuolo and Gribaldo 2010) and trimAl (Capella-Gutiérrez et al. 2009), and built with RAxML (Stamatakis 2014); manual inspection of gene trees to identify and remove paralogs and contaminants using ParaSorter (included with PhyloFisher); followed by re-alignment; trimming using the same procedure; and concatenation of the selected orthologs into a supermatrix. The final maximum likelihood tree was generated with IQ-TREE, using automatic model selection with ModelFinder, which selected the LG+R10 model, and 1000 ultrafast bootstraps (Nguyen et al. 2015) (File S12).

### Orthogroup generation and functional annotation

The studied proteomes were filtered with CD-HIT to remove redundancy (Fu et al. 2012) and screened for bacterial contamination using BLAST against eukaryotic and bacterial databases (Altschul et al. 1990). We used OrthoFinder2 with default parameter values, in order to cluster them into orthogroups, stored in the file “Orthogroups.tsv” (Emms and Kelly 2019).

We used eggNOG-mapper v2 with default parameter values to functionally annotate the proteomes of this project (Cantalapiedra et al. 2021). To infer the functional composition of each orthogroup, we collected all associated protein annotations and normalized their contributions by dividing each annotation by the total number of annotations within the orthogroup. If a protein had multiple functional annotations, its contribution was distributed equally among them by assigning a weight of 1 divided by the number of annotations.

*Rhizophagus irregularis* was excluded from this analysis due to its artifactual behavior (Figure S6), clustering closer to Metazoa than Fungi, which is not observed in Ocaña-Pallarès et al. although the same initial data and methodology was applied. *Rhizophagus irregularis*'s transcriptome did cluster with Fungi, which strengthened our suspicions on *Rhizophagus irregularis*'s genome. We hypothesize there was a mislabeling with *Rhizophagus irregularis*'s sequence, or that our newer version of eggNOG-mapper v2 gave us a different output (Cantalapiedra et al. 2021). Only Amoebozoa species with a high BUSCO value (>80%) were used in the clustering.

### Clustering of orthogroups and identification of overrepresented Pfam domain clans

We hierarchically clustered orthogroups by the number of representative genes present in each species in RStudio (RStudio Team 2020) with package pheatmap (Kolde 2010). Next, we automatically defined clusters and selected groups of orthogroups specific to each lineage by cutting the dendrogram, in order to generate phylogenetically coherent orthogroup clusters. To continue, we translated orthogroup presence within each detected cluster into Pfam clan content, allowing us to determine the relative abundance of Pfam clans. Finally, we combined the 20 most represented Pfam clans across all clusters to generate a heatmap of Pfam presence. To identify which Pfam clans were overrepresented in a given taxonomic group, we applied a simple heuristic: a Pfam clan was considered “overrepresented” if it (i) accounted for at least 0.5% of orthogroups within that cluster (ie it was sufficiently represented) and (ii) showed a minimum 1.4-fold higher presence compared to the second-highest cluster for that same Pfam clan (i.e. it was 40% more frequent.)

### Correspondence analyses

Correspondence analyses were done in RStudio (RStudio Team 2020) with the FactoMineR (Lê et al. 2008) package and the plots were constructed with the ggplot2 (Wickham 2011) package.

*Rhizophagus irregularis* was excluded from this analysis as described above. Several Amoebozoa species (*Micriamoeba sp. ATCC PRA-134*, *Squamamoeba japonica*, *Ovalopodium desertum*, *Mayorella cantabrigiensis*, *Thecamoeba sp. SK13-4B*, *Echinostelium bisporum*, *Mycamoeba gemmipara*, *Tychosporium acutostipes,* and *Pellita catalonica*) were also excluded due to a low BUSCO value (< 40%).

### Ancestral reconstruction

A proteomic dataset of 117 species representing Amorphea diversity and outgroups was used for ancestral reconstruction (36 of these species covering Fungi and Metazoa were obtained from Ocaña-Pallarès et al.) (File S11). Among these species, we include our *A. explorator* and *V. septentrionalis* assembled transcriptomes.

The input tree for the ancestral reconstruction was a manual merging of PhyloFisher and IQ-TREE's output (described above) and the Fungi and Metazoa topology from Ocaña-Pallarès et al. This topology was treated with Bppml (Guéguen et al. 2013), in order to estimate branch lengths and model parameters, with a configuration file, specifying a stationary process with a binary model and a gamma distribution of rate variation among sites, which was stated to remain homogeneous across all branches of the topology.

We ran our ancestral reconstructions with two different programs, in order to minimize and detect biased results generated by a strong effect of the methodology instead of the data (Gàlvez-Morante et al. 2024).

We ran Bppancestor (maximum likelihood, counts presence/absence of genes per orthogroup) with a configuration file specifying the most likely model estimated by Bppml. The used version was Bio++ version 3.0.0 (Guéguen et al. 2013).

We ran Notung (Wagner parsimony, counts number of ancestral genes per orthogroup) with the following command “notung -b batch_notung.txt --reconcile --events --parsable --absfilenames --log --silent --progressbar --speciestag prefix --phylogenomics”. The used version was Notung version 2.9.1 (K. Chen et al. 2000).

*Rhizophagus irregularis* was excluded from this analysis as described above.

## Supplementary Material

msag071_Supplementary_Data

## Data Availability

**Data**: Transcriptome reads and assemblies are available in GenBank under BioProject accession number PRJNA1257292. The ribosomal operon sequence (18S, ITS1, 5.8S, ITS2, 28S) sequence of BEAP0066 is available with accession number PV590302, and the 18S sequence of BEAP0079 is available with accession number PV584300. Supplementary files, videos, transcriptome assemblies, predicted proteomes and input data are available in FigShare with the DOI 10.6084/m9.figshare.28741229. The transcriptome assemblies available on GenBank differ from those on FigShare due to the automatic decontamination tools used by GenBank, which we believe have erroneously removed a number of valid contigs. As a result, we recommend using our FigShare assemblies, which retain these sequences, over the GenBank versions. **Code**: Scripts used in data analysis are available on GitHub at: https://github.com/beaplab/amorphea_evolution. **Cell cultures**: A cell culture containing *Apostamoeba explorator* strain BEAP0066 (and mixed bacteria) is publicly available and has been deposited in two culture collections: the Roscoff Culture Collection (RCC) with identifier RCC11540 and the Culture Collection of Algae and Protozoa (CCAP) with accession number CCAP2503/1. A cell culture containing *Vannella septentrionalis* strain BEAP0079 (and mixed bacteria) is publicly available and has been deposited in the Roscoff Culture Collection (RCC) with identifier RCC11541. A SEM stub for BEAP0066 has been deposited in the Marine Biological Reference Collections (CBMR) at the Institut de Ciències del Mar (ICM-CSIC, Barcelona, Spain) under the catalog/accession number ICMCBMR000695 (Guerrero et al., 2023).
