# Supplementary material for: The evolution of gene functional repertoire in Amorphea: divergent strategies across Amoebozoa, Fungi, and Metazoa
Source: Mol Biol Evol. 2026 May 20;43(5):msag071. doi: 10.1093/molbev/msag071 (PMC13191114; doi:10.1093/molbev/msag071)
Supplement: msag071_Supplementary_Data [file msag071_supplementary_data.zip › merged_supplementary_captions_and_figures.pdf]

Supplementary Video 1. BEAP0066, floating form. Video accelerated to x150 normal speed. Total time elapsed = 1.2 h.

Supplementary Video 2. BEAP0066, contact of subpseudopodia. Video accelerated to x150 normal speed. Total time elapsed = 1.6 h.

Supplementary Video 3. BEAP0066, double-amoeba. Video accelerated to x150 normal speed. Total time elapsed = 5.8 h.

Supplementary Video 4. BEAP0066, rings in solid medium. Video accelerated to x300 normal speed. Total time elapsed = 2.9 h.

Supplementary File 1. Classification of COG categories in dictyostelids according to their relative similarity to Fungi or Amoebozoa.

Supplementary File 2. Differentially expressed Pfam clans per taxonomic group.

Supplementary File 3. Genomes used for transcriptome decontamination.

Supplementary File 4. Sequences used in rRNA figures.

Supplementary File 5. 18S full alignment with the sequence mask.

Supplementary File 6. 18S trimmed alignment.

Supplementary File 7. Newick tree for Supplementary Figure 1.

Supplementary File 8. V4 full alignment with the sequence mask.

Supplementary File 9. V4 trimmed alignment.

Supplementary File 10. Newick tree for Supplementary Figure 2.

Supplementary File 11. Species used in the project with source and name abbreviation.

Supplementary File 12. Newick tree for Figure 1.

Supplementary File 13. Scanning electron microscopy additional images.

Supplementary File 14. Transmission electron microscopy additional images.

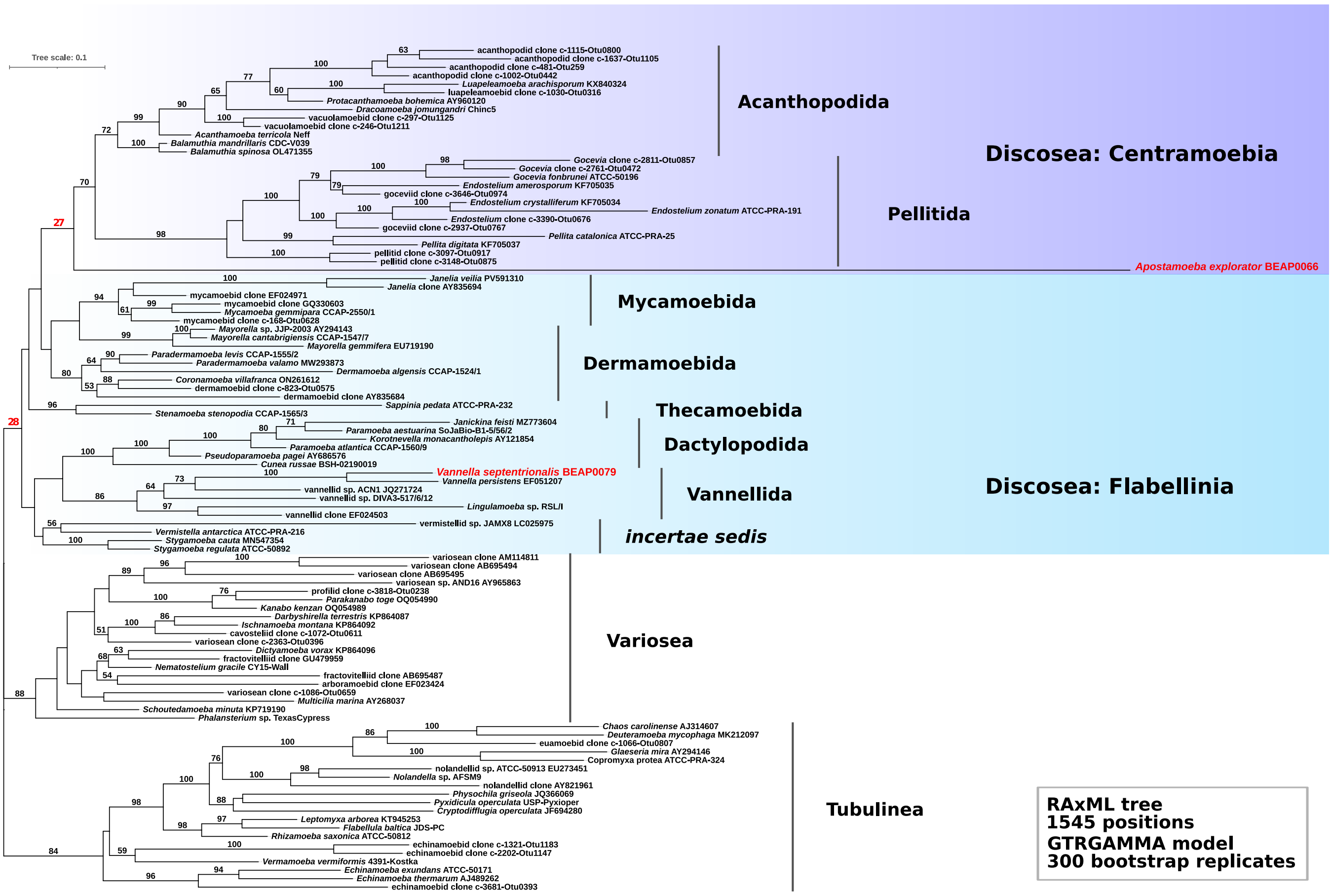

Supplementary Figure 1. Amoebozoa 18S phylogeny. Phylogenetic tree of Amoebozoa 18S diversity based on 1545 positions. Phylogeny reconstructed with RAxML using the GTRGAMMA model of sequence evolution with 300 bootstrap inferences. We excluded bootstrap support values below 50%, with the exception of the support value for Acapostellia (27%) and Discosea (28%). *Apostamoeba explorator* (BEAP0066) and *Vannella septentrionalis* (BEAP0079) are highlighted in red. Himatismenida sequences were excluded from the tree, as they were attracted to Tubulinea. The sequences used for the construction of this tree, together with their origins, are listed in the Supplementary File 4. The alignment and tree files are Supplementary File 5, Supplementary File 6 and Supplementary File 7.

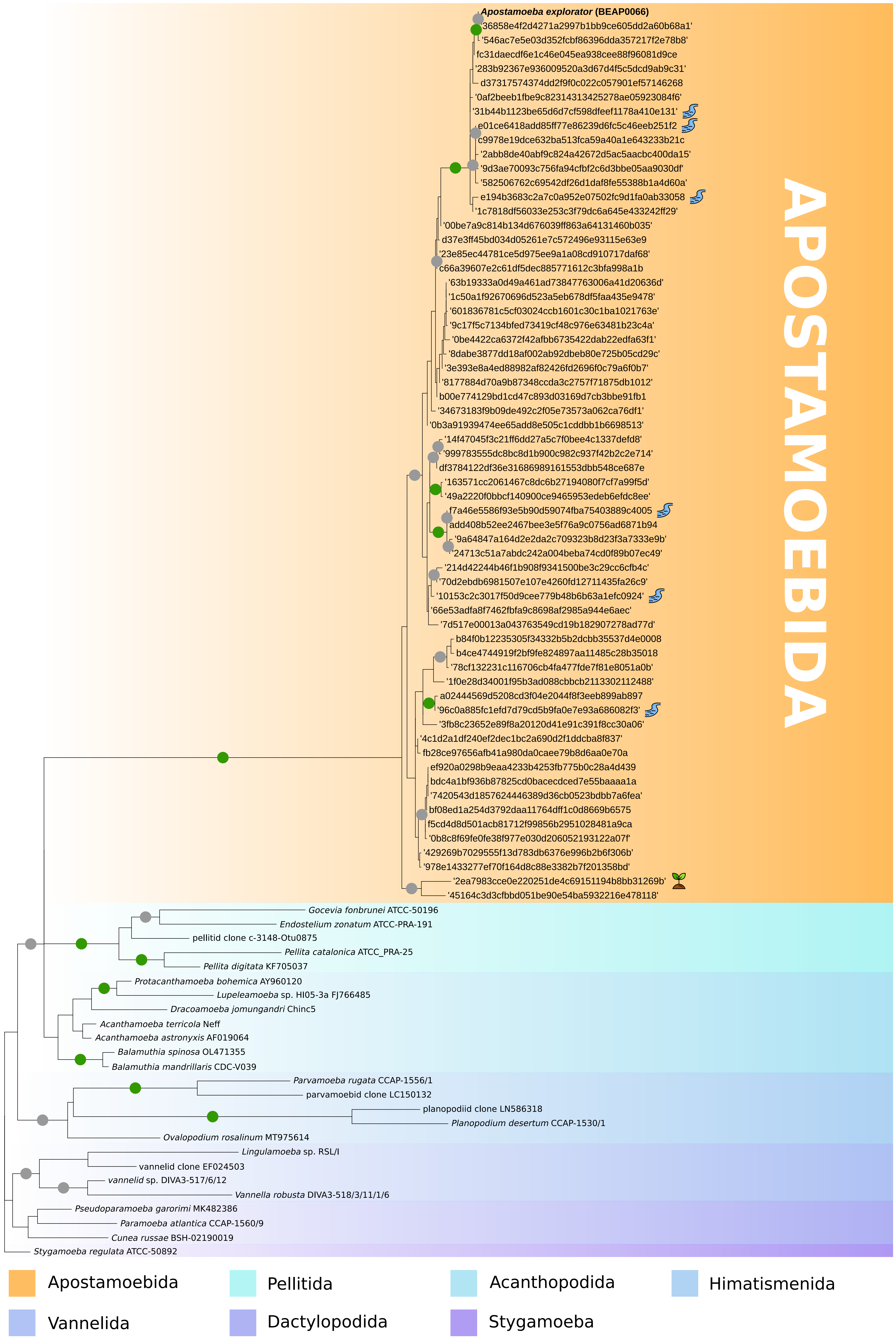

Supplementary Figure 2. *Apostamoebida environmental* V4 diversity. Phylogenetic tree of Apostamoebida diversity based on V4 18S region (330 positions). Phylogeny reconstructed with RAxML using the GTRCAT model of sequence evolution with 1000 rapid bootstrap inferences. Sequences with a GenBank identifier were retrieved from NCBI, one pellitid sequence was extracted from the long-read amplicon dataset of Jamy et al. (Jamy et al., 2020), and the remaining sequences were retrieved from transcriptomes in EukProt (Richter et al., 2022). Green dots represent a node support over 95, while grey dots represent a node support over 75. Support for the node separating Apostamoebida from other groups is 100. All Apostamoebida representatives are found in marine samples, except '2ea7983cce0e220251de4c69151194b8bb31269b', which was found exclusively in soil samples. ASVs marked with a river symbol were found both in marine and freshwater samples. See Supplementary File 10 for the phylogenetic tree with all outrgroup species represented.

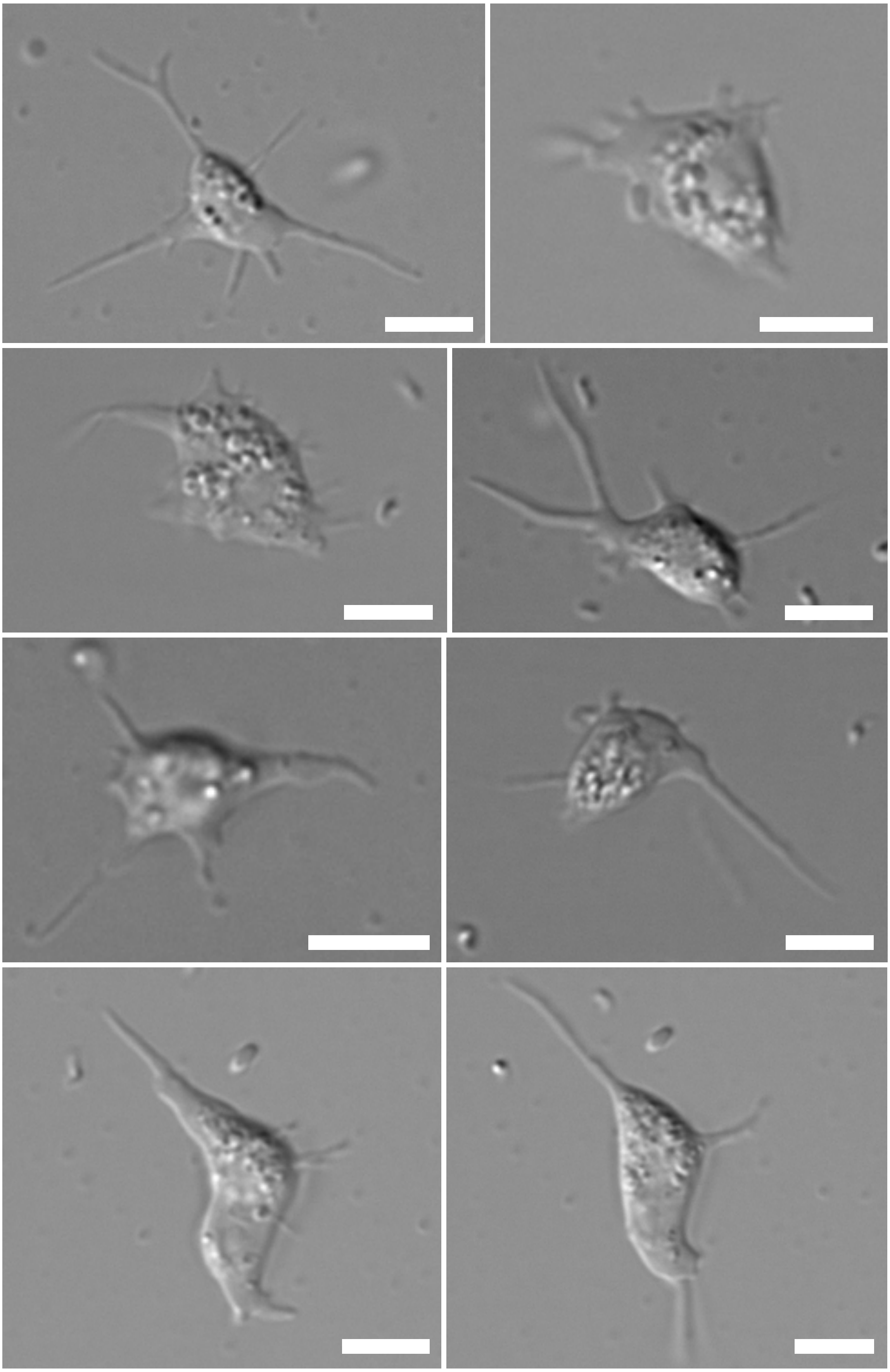

Supplementary Figure 3. Morphology of *Apostamoeba explorator* through Differential interference contrast (DIC) images. Differential interference contrast (DIC) images of *Apostamoeba explorator* locomotive cells. Scale bars in all panels = 5 µm.

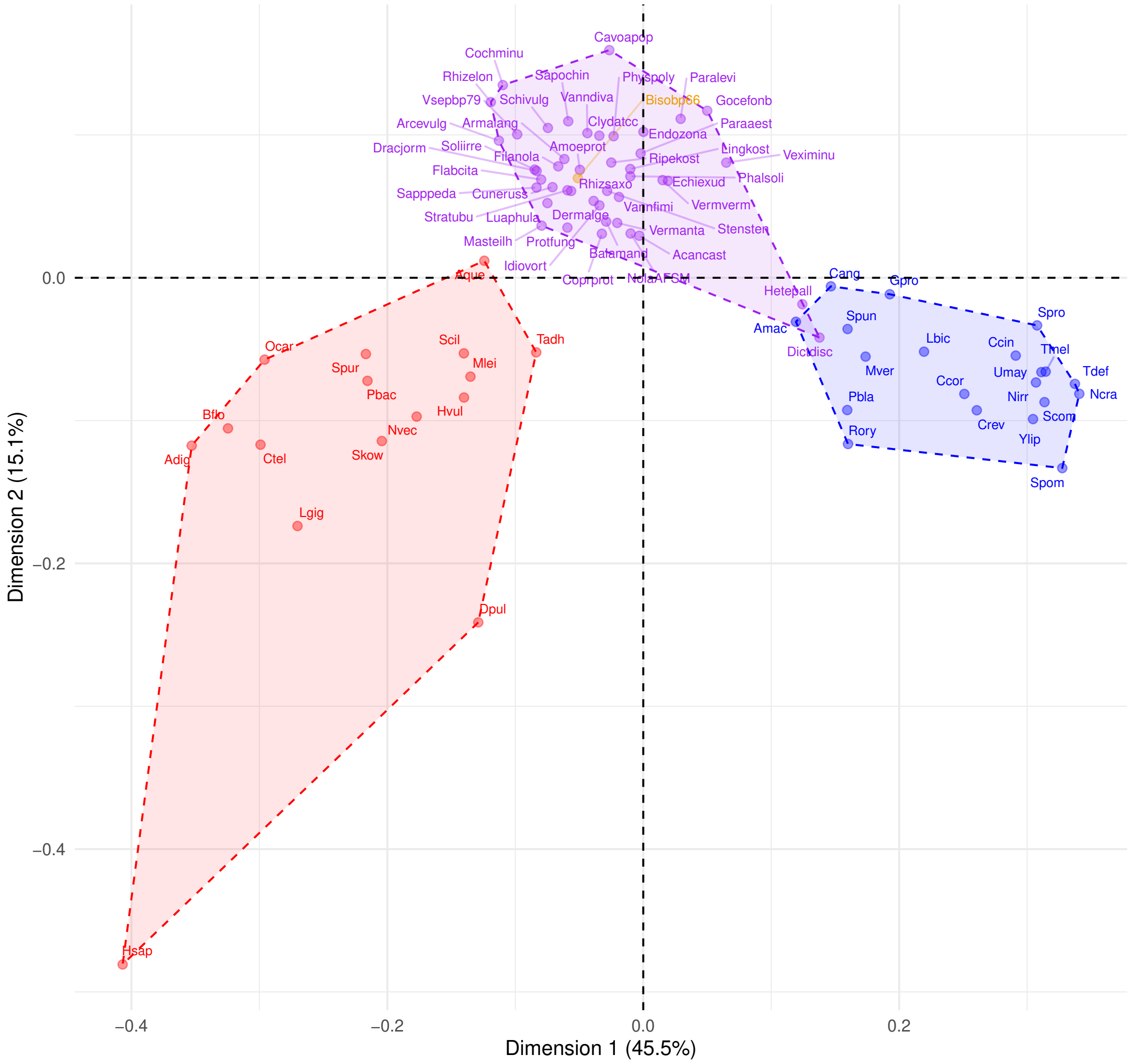

Taxonomic group

- Amoebozoa
- Ancestor–Notung
- Fungi
- Metazoa

COG categories

A = RNA processing and modification
B = Chromatin structure and dynamics
C = Energy production and conversion
D = Cell cycle control, cell division, chromosome partitioning
E = Amino acid transport and metabolism
F = Nucleotide transport and metabolism
G = Carbohydrate transport and metabolism
H = Coenzyme transport and metabolism
I = Lipid transport and metabolism
J = Translation, ribosomal structure, and biogenesis
K = Transcription
L = Replication, recombination, and repair
M = Cell wall/membrane/envelope biogenesis
N = Cell motility
O = Posttranslational modification, protein turnover, chaperones
P = Inorganic ion transport and metabolism
Q = Secondary metabolites biosynthesis, transport, and catabolism
R = General function prediction only
S = Function unknown
T = Signal transduction mechanisms
U = Intracellular trafficking, secretion, and vesicular transport
V = Defense mechanisms
W = Extracellular structures
Y = Nuclear structure
Z = Cytoskeleton

Supplementary Figure 4. Correspondence Analyses (CA) of Clusters of Orthologous Groups (COG) functional category compositions of the gene complements of Amoebozoa (purple), Metazoa (red) and Fungi (blue) with all species labeled. First two dimensions of CA. Each point represents the gene complement of a single species (from a genome or a transcriptome; Supplementary figure 6B shows that data source does not have a significant impact). Correspondence between the species abbreviations and their full names can be found in Supplementary File 11.

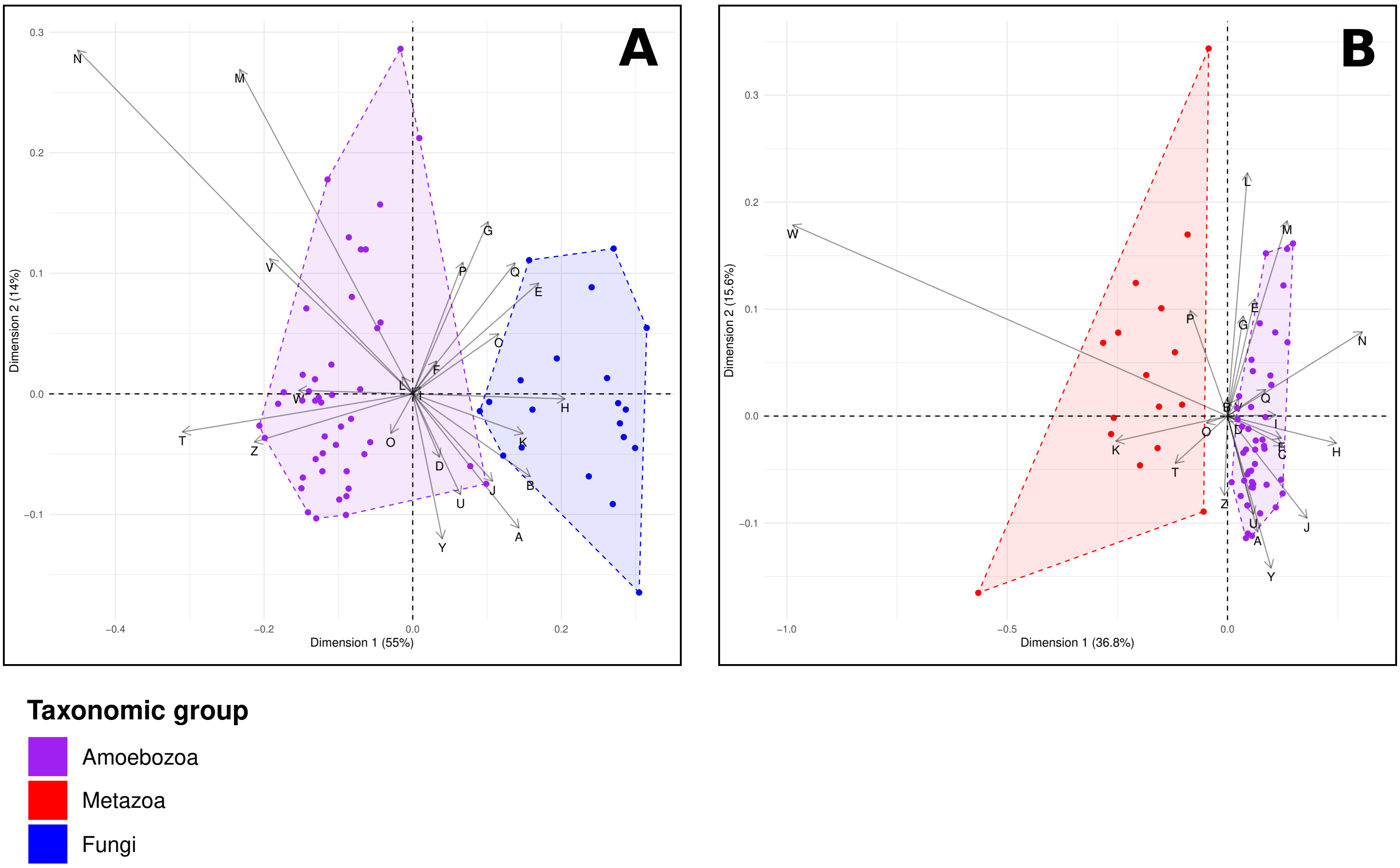

Supplementary Figure 5. Correspondence Analyses (CA) of Clusters of Orthologous Groups (COG) functional category compositions of the gene complements of Amoebozoa against either Fungi or Metazoa. First two dimensions of CA. Each point represents the gene complement of a single species (from a genome or a transcriptome; Supplementary figure 6B shows that data source does not have a significant impact). **A.** Amoebozoa (purple) compared with Fungi (Blue). **B.** Amoebozoa (Purple) compared with Metazoa (Red).

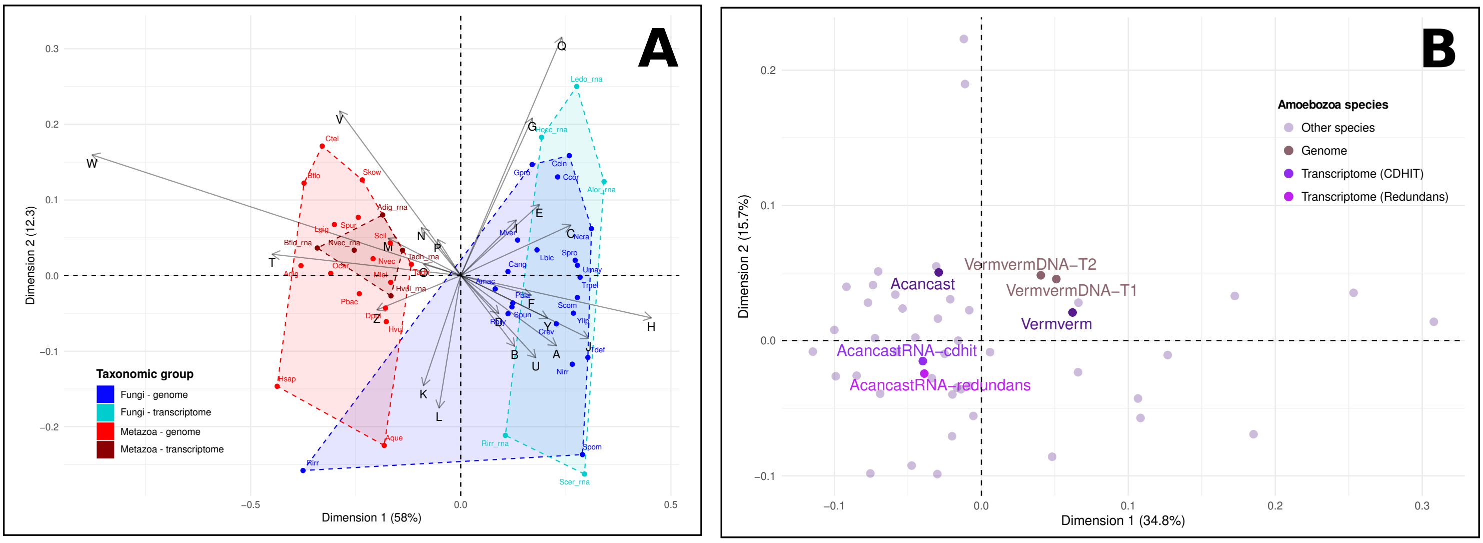

Supplementary Figure 6. Correspondence Analyses (CA) of Clusters of Orthologous Groups (COG) functional category compositions of gene complements estimated with different methods. A. Metazoa (red) and Fungi (blue) genomic and transcriptomic gene complements are compared and cluster together. Five transcriptomes per taxonomic group were selected, each with a genomic counterpart from our study (same species or closely related). All used datasets are publicly available. The genome complement of Rirr (*Rhizopus irregularis*), which was excluded from our other analysis, does not cluster with other fungi, although its transcriptome complement is less of an outlier. **B.** Amoebozoa genomic (brown) and transcriptomic (purple) gene complements compared with their counterparts. *Acanthamoeba castellani* (Acancast) transcriptome was processed separately with either CD-HIT or Redundans, in order to reduce redundancy and to assess the equivalence of these programs, and compared with its genome (originally used in the project). Genes in the *Vermamoeba vermiformis* (Vermverm) genome were predicted with EukMetaSanity and compared with its transcriptome (originally used in the project).

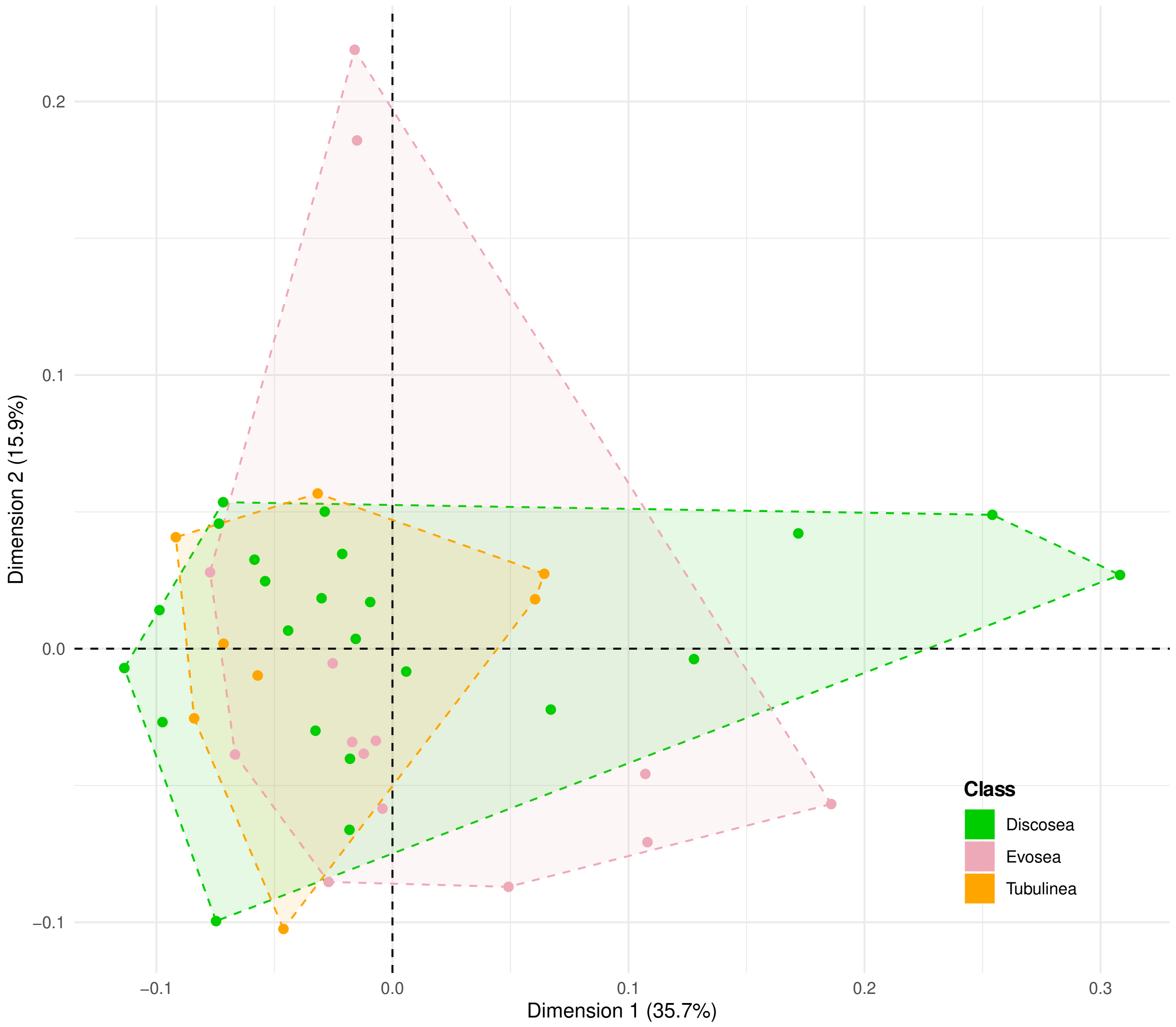

Supplementary Figure 7. Correspondence Analyses (CA) of Clusters of Orthologous Groups (COG) functional category compositions of the gene complements of groups within Amoebozoa. Discosea (green), Tubulinea (orange) and Evosea (pink). First two dimensions of CA. Each point represents the gene complement of a single species (from a genome or a transcriptome; Supplementary figure 6B shows that data source does not have a significant impact).

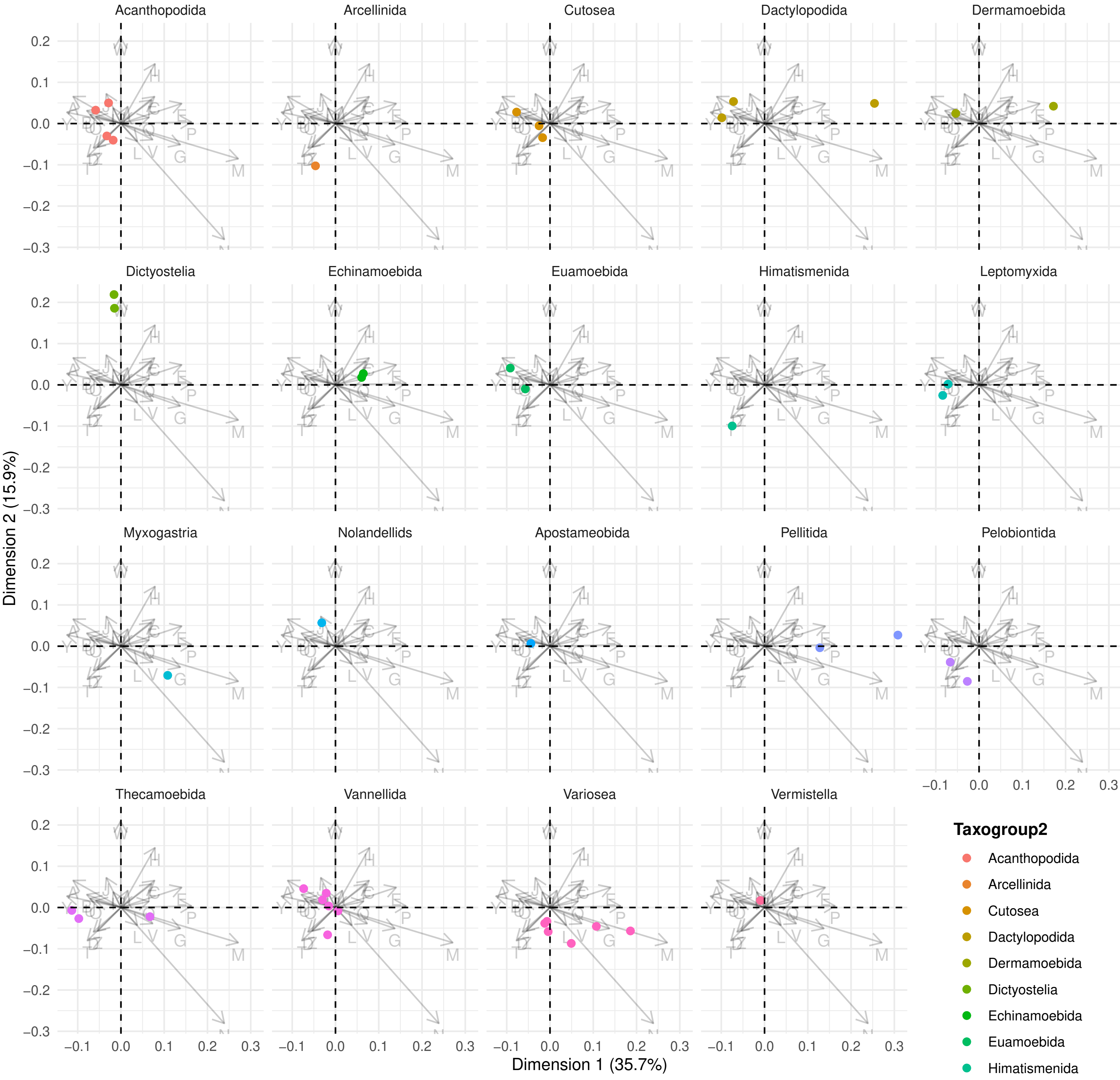

Supplementary Figure 8. Correspondence Analyses (CA) of Clusters of Orthologous Groups (COG) functional category compositions of the gene complements displayed for individual groups within Amoebozoa. Taxonomic groupings are taken from UniEuk, at the Taxogroup2 level (Berney et al., 2017). A single CA was performed. Different panels highlight members of each Taxogroup2, in color. First two dimensions of CA. Each point represents the gene complement of a single species (from a genome or a transcriptome; Supplementary figure 6B shows that data source does not have a significant impact).

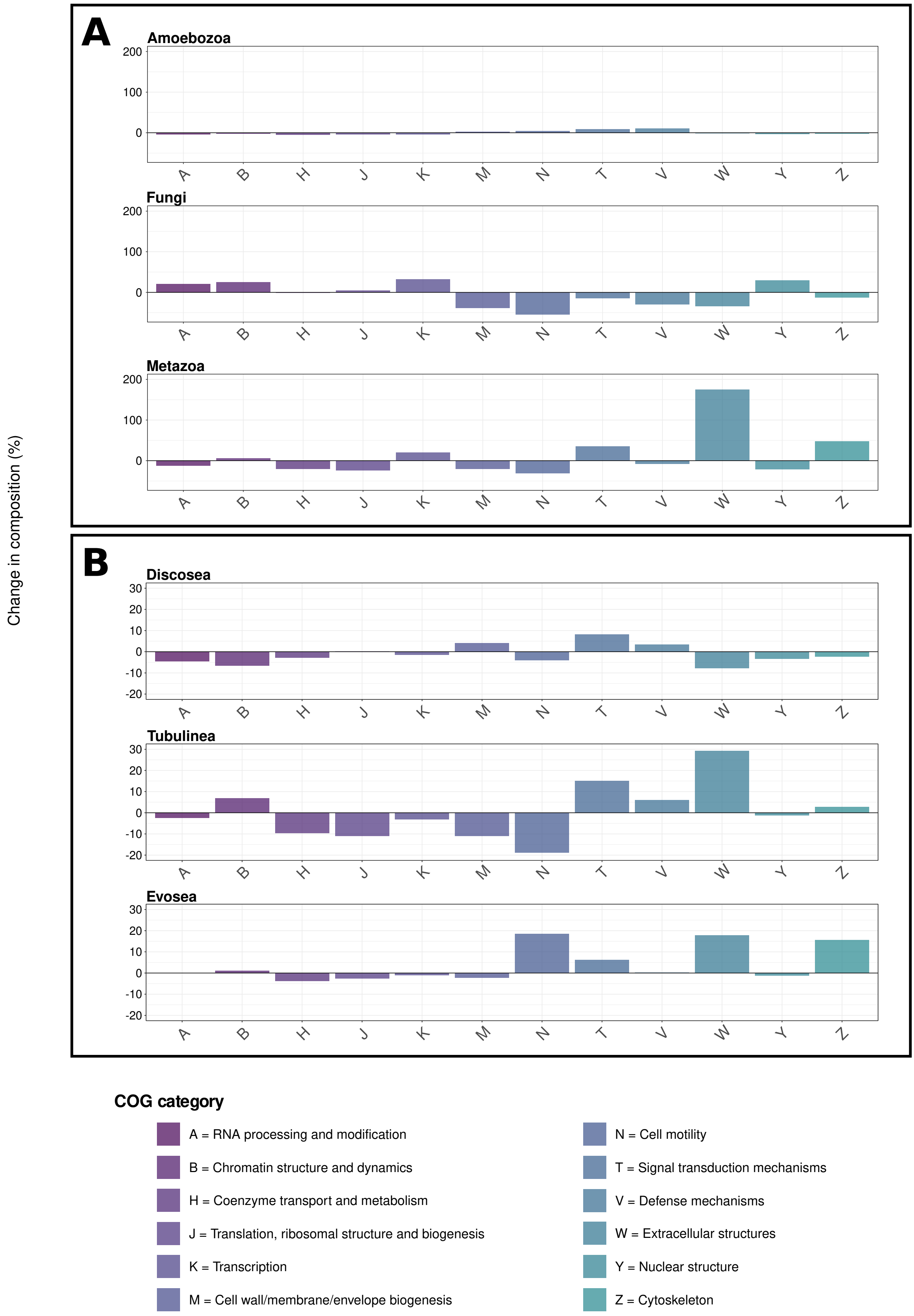

Supplementary Figure 9. Reconstructed ancestral gains and losses in Clusters of orthologous groups (COG) functional category composition. A. Changes on the stem lineage leading from Amorphea to the last common ancestors of Amoebozoa, Fungi or Metazoa. **B.** Changes on the stem lineage leading from Amoebozoa to the last common ancestors of Discosea, Tubulina or Evosea. Only a subset of COG categories are represented in the figure, specifically those with a change greater than 20% from the Amorphea ancestor to either Amoebozoa, Fungi, or Metazoa.

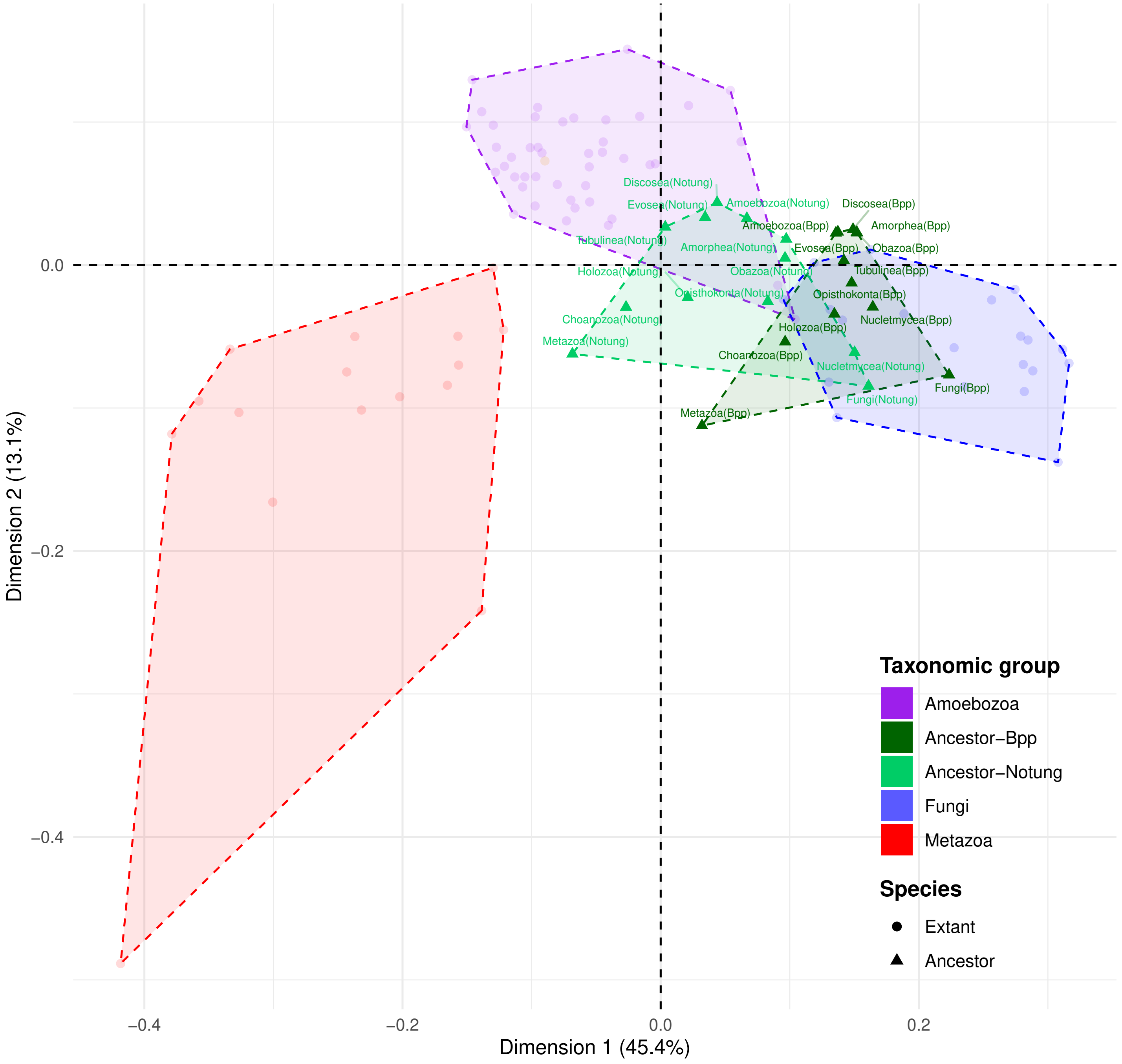

Supplementary Figure 10. Correspondence Analyses (CA) of Clusters of Orthologous Groups (COG) functional category compositions of the gene complements of Amoebozoa, Metazoa, Fungi and their most recent common ancestors. First two dimensions of CA. Each point represents the gene complement of a single species (from a genome or a transcriptome; Supplementary figure 6B shows that data source does not have a significant impact). Gene complements of most recent common ancestors were inferred by orthologous gene identification (OrthoFinder2) and either Wagner parsimony (Notung) or maximum likelihood (Bpp). Wagner parsimony estimates ancestral counts for each OrthoFinder2 gene family, whereas maximum likelihood with Bpp estimates ancestral presence or absence.

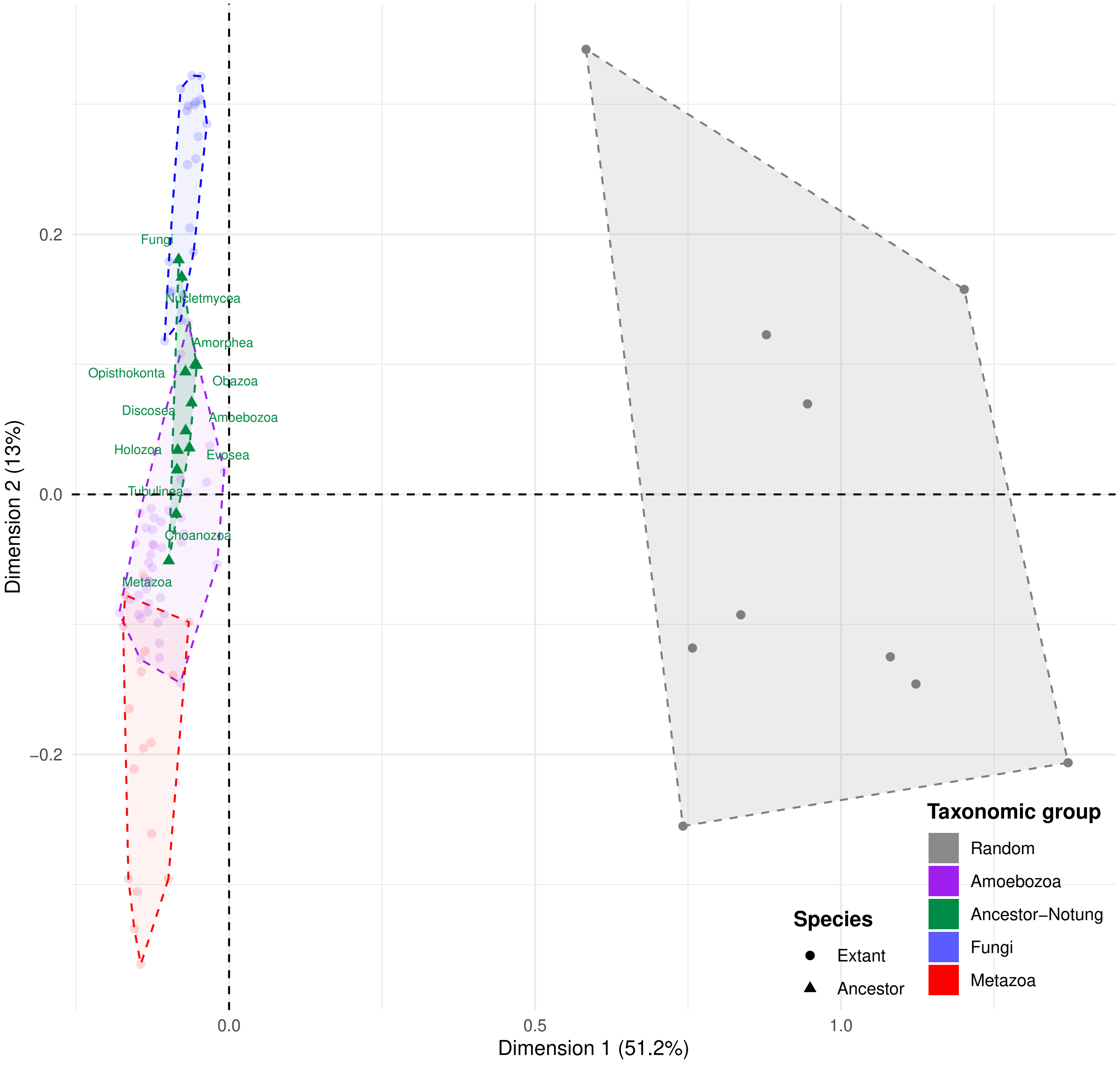

Supplementary Figure 11. Correspondence Analyses (CA) of Clusters of Orthologous Groups (COG) functional category compositions of the gene complements of Amoebozoa (purple), Metazoa (red), Fungi (blue) and their most recent common ancestors (green), together with randomly generated gene complements (grey). First two dimensions of CA. Each point represents the gene complement of a single species (from a genome or a transcriptome; Supplementary figure 6B shows that data source does not have a significant impact). Most recent common ancestors with gene complements were inferred by orthologous gene identification (OrthoFinder2) followed by Wagner parsimony (Notung). The points in the different random sets clusters are distant from the species we analyzed.
